# Uropathogenic *Escherichia coli* Infection Compromises the Blood-Testis Barrier by Disturbing mTORC1-mTORC2 Balance

**DOI:** 10.3389/fimmu.2021.582858

**Published:** 2021-02-19

**Authors:** Yongning Lu, Miao Liu, Nicholas J. Tursi, Bin Yan, Xiang Cao, Qi Che, Nianqin Yang, Xi Dong

**Affiliations:** ^1^ Reproductive Medicine Centre, Zhongshan Hospital, Fudan University, Shanghai, China; ^2^ Department of Biology, University of Pennsylvania, Philadelphia, PA, United States

**Keywords:** blood-testis barrier, uropathogenic *E. coli*, orchitis, mammalian target of rapamycin, male infertility

## Abstract

The structural and functional destruction of the blood-testis barrier (BTB) following uropathogenic *E. coli* (UPEC) infection may be a critical component of the pathologic progress of orchitis. Recent findings indicate that the mammalian target of the rapamycin (mTOR)-signaling pathway is implicated in the regulation of BTB assembly and restructuring. To explore the mechanisms underlying BTB damage induced by UPEC infection, we analyzed BTB integrity and the involvement of the mTOR-signaling pathway using *in vivo* and *in vitro* UPEC-infection models. We initially confirmed that soluble virulent factors secreted from UPEC trigger a stress response in Sertoli cells and disturb adjacent cell junctions *via* down-regulation of junctional proteins, including occludin, zonula occludens-1 (ZO-1), F-actin, connexin-43 (CX-43), β-catenin, and N-cadherin. The BTB was ultimately disrupted in UPEC-infected rat testes, and blood samples from UPEC-induced orchitis in these animals were positive for anti-sperm antibodies. Furthermore, we herein also demonstrated that mTOR complex 1 (mTORC1) over-activation and mTORC2 suppression contributed to the disturbance in the balance between BTB “opening” and “closing.” More importantly, rapamycin (a specific mTORC1 inhibitor) significantly restored the expression of cell-junction proteins and exerted a protective effect on the BTB during UPEC infection. We further confirmed that short-term treatment with rapamycin did not aggravate spermatogenic degeneration in infected rats. Collectively, this study showed an association between abnormal activation of the mTOR-signaling pathway and BTB impairment during UPEC-induced orchitis, which may provide new insights into a potential treatment strategy for testicular infection.

## Introduction

Approximately 6% to 15% of male infertility is attributed to infections or inflammation of the urogenital tract (1). Compared with the impacts of urethritis and prostatitis, the sequelae of epididymitis or orchitis are more likely to result in reduced fertility—as spermatogenetic, sperm maturational, and storage microenvironments may be directly exposed to pathogens and inflammatory products. Uropathogenic *Escherichia coli* (UPEC) is one of the major pathogens involved in ascending, non-sexually transmitted epididymo-orchitis (2–4). The elimination of invading pathogens by antibiotic administration is currently the major standardized therapy prescribed in acute bacterial epididymo-orchitis (4–6). Unfortunately, in most cases antibiotic treatment alone cannot guarantee the full restoration of fertility due to permanent tissue damage or immunologic impairment within these organs (2, 6, 7). A better understanding of the mechanisms by which uropathogen-related orchitis disturbs testes functions may assist clinicians in developing better treatment strategies for fertility protection.

Investigations into the immunopathological mechanisms implicated in orchitis are particularly noteworthy considering the role of testicular immune privilege with respect to spermatogenic conservation. The blood-testis barrier (BTB) is one of the most well studied functional tissue barriers, and contributes to maintaining the special immune microenvironment in the testis (8, 9). This barrier is located at the interface of juxtaposed Sertoli cells, and is comprised of multiple junctional complexes—including tight junctions (TJs), gap junctions, adhesion junctions, and desmosomes (10, 11). The BTB sequesters meiotic spermatocytes and antigen-expressing, post-meiotic spermatids away from the immune system (10). Despite being one of the tightest blood-tissue barriers, the BTB undergoes restructuring during stage VIII-XI of the seminiferous epithelial cycle to support preleptotene spermatocytes that transit through this barrier (9, 12). In this process, a “new” BTB is assembled behind the transiting spermatocytes while the “old” BTB noted above is disassembled (10). The remodeling mechanisms of the BTB guarantee immunologic barrier integrity and prevent the development of autoimmune responses against germ cells.

Although the BTB restructuring mechanism has yet to be fully understood, accumulating evidence suggests that the mammalian target of rapamycin (mTOR) contributes to the regulation of this physiologic process during the epithelial cycle of spermatogenesis (13, 14). Two functionally and structurally distinct mTOR-signaling complexes (mTOR complex 1 [mTORC1] and 2 [mTORC2]) are formed depending upon whether their partner proteins Raptor (regulatory-associated protein of mTOR) or Rictor (rapamycin-insensitive companion of mTOR) combine with the core component (15). These two mTOR complexes exert their antagonistic effects to facilitate the transition of preleptotene spermatocytes across this immunologic barrier, as well as to maintain structural and functional integrity of the BTB. Recent evidence also indicates that mTORC1 promotes BTB disassembly by inducing redistribution and endocytosis of junctional proteins (16, 17). Conversely, mTORC2 contributes to the maintenance of BTB integrity and the assembly of a “new” barrier (18). A delicate mTORC1-mTORC2 balance is thus critical for the preservation of BTB function. In the context of pathologic situations, it was found that viral infection or inflammation caused BTB impairment by triggering the release of cytokines, such as TNF-α, and activating inflammatory-signaling pathways, including the p38 mitogen-activated protein (MAP) kinase pathway (19, 20). However, the role of the mTOR pathway remains arcane with respect to potential alterations of the BTB during bacterially-induced orchitis.

Using *in vivo* and *in vitro* infection models, our previous studies showed that the inflammatory response in the testis and damage to the Sertoli cells was caused by UPEC. In the present study, we further elucidated the dynamic alterations of the BTB in UPEC-induced orchitis and explored the possibility of preserving BTB structure and function by manipulating the balance between mTORC1 and mTORC2.

## Materials and Methods

### Bacterial Propagation and Preparation of Culture Supernatant

The uropathogenic *Escherichia coli* (UPEC) strain CFT073 was purchased from American Type Culture Collection (ATCC, Gaithersburg, Maryland, USA) and propagated as previously described (21). UPEC was propagated overnight on agar plates. A single bacterial clone was then inoculated in lysogeny broth (LB) agar medium until grown to early exponential phase (OD600 = 0.4~0.8) at 37°C in a shaking incubator. The viable bacterial concentration was estimated using standard growth curves. Bacteria were centrifuged at 4500×g for 8 min at room temperature and the pellet was washed once with PBS and resuspended in DMEM/F12 medium or sterile saline. The concentrated bacterial suspension was then adjusted to 1×10^9^ colony-forming units (CFU) per milliliter. For *in vivo* experiments, the bacterial suspension was diluted with sterile saline to achieve 1×10^6^ CFU of bacteria in 100 µl. For Sertoli cell *in vitro* stimulation, the bacterial suspension was subsequently centrifuged at 4500×g for 10 minutes, and the collected supernatant was ultimately filtered with 0.1-μm filters before use.

### Rat Orchitis Model

All experiments were approved by the Ethical Committee of Zhongshan Hospital and performed in accordance with the Guide for the Care and Use of Laboratory Animals. Nine-week-old male Sprague-Dawley (SD) rats were purchased from CAVENS (Changzhou, Jiangsu, China) and housed in 12 h light/12 h dark standard conditions, with water and food provided *ad libitum* for at least 1 week prior to experimentation. The orchitis model was modified on the basis of previous studies (21). Adult rats (270–300 g) were injected intramuscularly with Zoletil™ 20 (Virbac, Carros, France; 50 mg/kg of body weight) for anesthetization. Fifty microliters of UPEC bacterial suspension per side (0.5×10^6^ CFU) was percutaneously injected into both testes of rats in the infected groups using 30-gauge needles. The same volume of 0.9% saline was applied as a control. In the rapamycin-treated groups, different doses of rapamycin (2, 5, or 10 mg/kg/day) were administrated intraperitoneally on day 7 post infection for seven days based on a previous report (22). The remaining infected rats received the same amount of vehicle (5% ethanol +5% PEG 400 + 5% Tween 80 + 85% saline) as the vehicle-treated group. Euthanasia of animals was conducted at certain time-points. After recording body and testis weights, serum, and testes were collected for further experiments. In each group there were 12 (control group)–15 (infected and rapamycin-treated groups) rats used for all experiments. The bilateral testes from six animals in each group were fixed for transmission electron microscopy and paraffin-section assessments. The testes of the remaining animals were assigned for biotin assay, frozen sections, and western blotting analysis. For the biotin penetration assay, 100 μl of EZ-Link™ sulfo-NHS-LC-biotin was loaded into the testes and organs were harvested as described in the literature (23). Rats that were intraperitoneally (i.p.) injected with CdCl_2_ (3 mg/kg body weight) served as positive controls, and testes were collected after three days.

### Sertoli Cell Culture and Treatment With Bacterial Supernatants

The mouse Sertoli cell line TM4 was purchased from ATCC and cultured in complete growth medium according to ATCC instructions. Primary Sertoli cells were isolated from the testes of 4-week-old male Sprague-Dawley (SD) rats as previously described (24). The purity of the Sertoli cells was determined to be >95% by immunofluorescence evaluation using antibodies against vimentin (**Supplementary Figure 1**). Sertoli cells were cultured in serum-free DMEM/F12 (ThermoFisher Scientific, Asheville, NC, USA) supplemented with penicillin/streptomycin. In the infected groups, Sertoli cells were exposed to different amounts of filtered UPEC supernatant (40 or 60 μl per 200 μl of culture medium) with or without rapamycin (Selleckchem, Houston, TX, USA) and cultured for 24 or 40 hours. Rapamycin dissolved in DMSO (at a storage concentration of 10 mM) was used to treat cells at a final concentration of 0.1 nM or 0.1 μM based on a previous report (17, 25) and our pilot study. Culture medium was replaced every 10–12 hours.

### Detection of Anti-Sperm Antibodies

Serum samples were collected from animals at 3 months post infection and sperm-bound total immunoglobulins were detected using an ELISA kit (BIOSH Biotechnology Limited Company, Shanghai, China), following the manufacturer’s instructions. Fifty microliters of each serum sample—as well as positive/negative control solutions provided in the kit—was added to a 96-well assay plate coated with specific capture antigens for anti-sperm antibodies and incubated for 30 minutes. Horseradish peroxidase (HRP)-conjugated detection antibody was subsequently added and tetramethylbenzidine served as the substrate. We detected the optical density (OD) at a wavelength of 450 nm using a microplate reader (ThermoFisher Scientific, Asheville, NC, USA).

### Electron Microscopy

To reveal changes in BTB ultrastructure after infection, 1 mm^3^ of testicular tissues were fixed in 2.5% glutaraldehyde/0.1 M sodium phosphate (pH, 7.35) and ultra-thin sections (70 nm) were prepared for subsequent evaluation using transmission electron microscopy (HITACHI HT7800, TOKYO, Japan) at 80 kV. The connections between cultured TM4 cells were observed with scanning electron microscopy (SEM) using cells fixed on glass coverslips.

### Histologic Assessment of the Testes

For assessment of testicular histopathology, sections (4 μm in thickness) of modified Davidson’s fluid-fixed and paraffin-embedded testis were stained with hematoxylin and eosin as previously described (21). Three sections from different regions of each testis were then used for histopathologic evaluation. The percentage of abnormal seminiferous tubules in cross sections was calculated by assessing over 200 tubules in each testis.

### Immunofluorescence Staining

Frozen-tissue sections of testes (8 µm in thickness) and Sertoli cells grown on glass coverslips were fixed with ice-cold methanol and permeabilized with 0.1% Triton-X 100. Immunofluorescence analyses were performed as described by Bhushan et al (24). Sections of testes were blocked with 5% BSA +5% goat serum for 1 hour at room temperature. Primary antibodies were incubated at 4°C overnight, followed by rinsing 3 times with Tris-buffered saline (TBS). Incubation with anti-rabbit or anti-mouse secondary antibody was performed in the dark for 1 hour, and CoraLite^®^594-conjugated phalloidin was applied to identify F-actin filaments. Slides were mounted with antifade mounting medium using Hoechst 33342 (P0133, Beyotime Biotechnology, Shanghai, China) for nuclear staining prior to assessment with an inverted laser scanning confocal microscope (TiE-A1 plus, Nikon-instruments, Tokyo Metropolis, Japan). The list of antibodies used is provided in the **Supplementary Materials**.

### Western Immunoblotting Analysis

Different groups of Sertoli cells and testicular tissue homogenates were lysed with RIPA buffer (P0013B, Beyotime Institute of Biotechnology, Shanghai, China) supplemented with a proteinase-inhibitor cocktail (Roche, Basel, Switzerland). Thirty micrograms of protein from each sample was resolved using 4–12% or 8% SDS-PAGE and subsequently transferred onto 0.22-μm PVDF membranes (at 200 mA for 3.5 hours). The nonspecific binding sites were blocked by incubating the membranes in 5% BSA in TBS with 0.1% Tween (TBS-T) for 1 hour. The membranes were subsequently incubated with primary antibodies in blocking solution overnight at 4°C and then rinsed 3 times with TBS-T. Membranes were ultimately incubated for 1 hour with anti-mouse or anti-rabbit antibody conjugated with HRP and then rinsed 3 times with TBS-T. Protein expression was measured as previously reported (24). The antibodies used are listed in the **Supplementary Materials**. ImageJ (https://imagej.nih.gov) was used to quantify the intensity of bands after western blotting. Uncropped original figures of all blots shown in this manuscript are presented in **Supplementary PowerPoint**.

### Statistical Analysis

Measurement data are presented as mean ± standard deviation (SD), and statistical significance between/among groups was analyzed using Student’s *t* test, one-way ANOVA, or Mann–Whitney U test (2-sided). Differences were considered to be statistically significant when *p*<0.05 (we used *p<0.05, **p<0.01, and ***p<0.001).

## Results

### BTB Alterations and Anti-Sperm Antibody Detection in UPEC-Infected Rats

**Figure 1:**
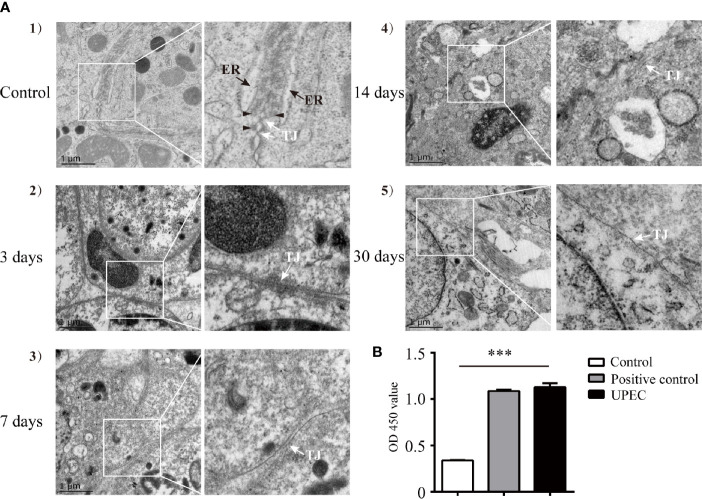
UPEC infection disrupts BTB structure and results in an elevation of serum anti-sperm antibodies in an orchitis rat model. A bacterial suspension (0.5×10^6^ CFU/50 μl) or 50 μl of saline was bilaterally injected into testes of 10–11-week-old SD rats. **(A)** Animals were sacrificed at the indicated time-points and BTB structure in the testis was observed using TEM. Scale bar, 1 μm. White arrow, TJ (tight junction); black arrow, ER (endoplasmic reticulum); black arrowhead, F-actin bundles. The photographs in the right column are the enlarged images of the white box regions. **(B)** Serum samples were collected from rats after three months, and anti-sperm antibodies in serum were detected with ELISA kits. Histograms show quantitative results, and *p* value was determined with ANOVA; ***p<0.001.

**Figure 2:**
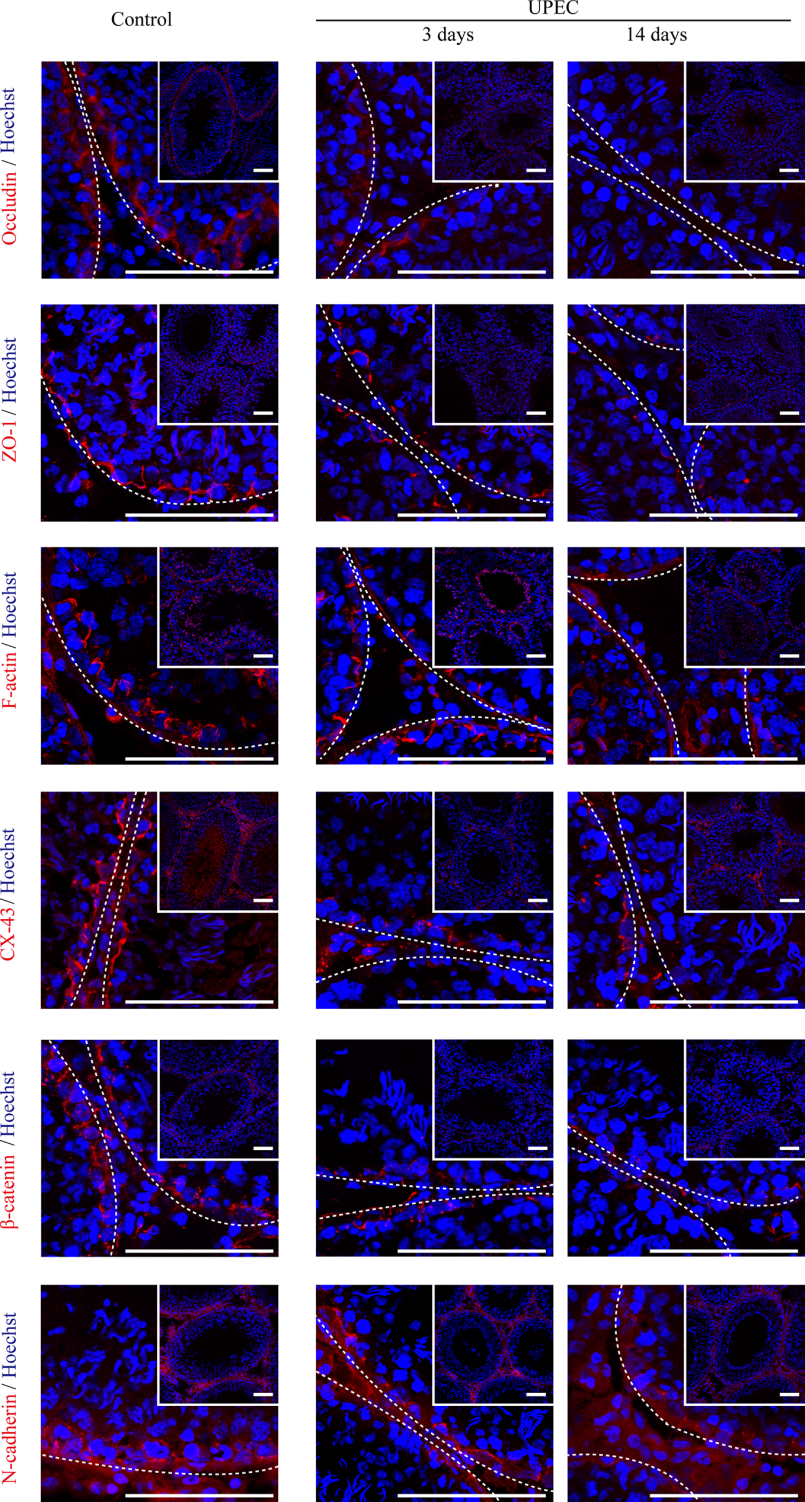
UPEC infection down-regulates the expression of cell-junction proteins in the testes of orchitis-model rats. In the orchitis model, rats were sacrificed at the indicated times, and we evaluated the expression of cell-junction proteins in seminiferous epithelium using immunofluorescence staining under an inverted laser scanning confocal microscope. Red, cell-junction proteins; blue, Hoechst 33342 nuclear staining. The images in the insets are the same testes cross-sections at lower magnification (scale bar, 100 μm). The white broken lines indicate the relative location of the basement membrane adjacent to the BTB of the seminiferous tubules.

**Figure 3:**
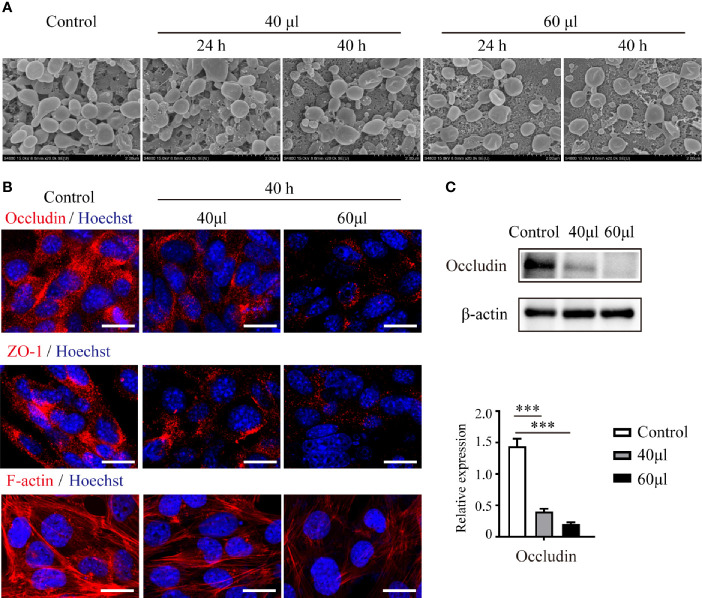
UPEC-secreted virulence factors in cell culture supernatant disturb Sertoli cell connections and down-regulate the expression of cell-junction proteins. Sertoli cells treated with the indicated amounts of supernatant from UPEC bacterial cultures for 24 or 40 hours. **(A)** TM4 cellular connections were observed using SEM. Scale bar, 2 μm. **(B)** Expression of cell-junction proteins in primary Sertoli cells were evaluated using immunofluorescence and observed under confocal microscopy. Scale bar, 20 μm. **(C)** The expression levels of occludin in primary Sertoli cells were evaluated using immunoblotting analysis, and representative blots are depicted. Data are presented as mean ± SD from triplicate experiments. Histograms shows quantitative results and *p* values were determined by ANOVA; ***p < 0.001.

**Figure 4:**
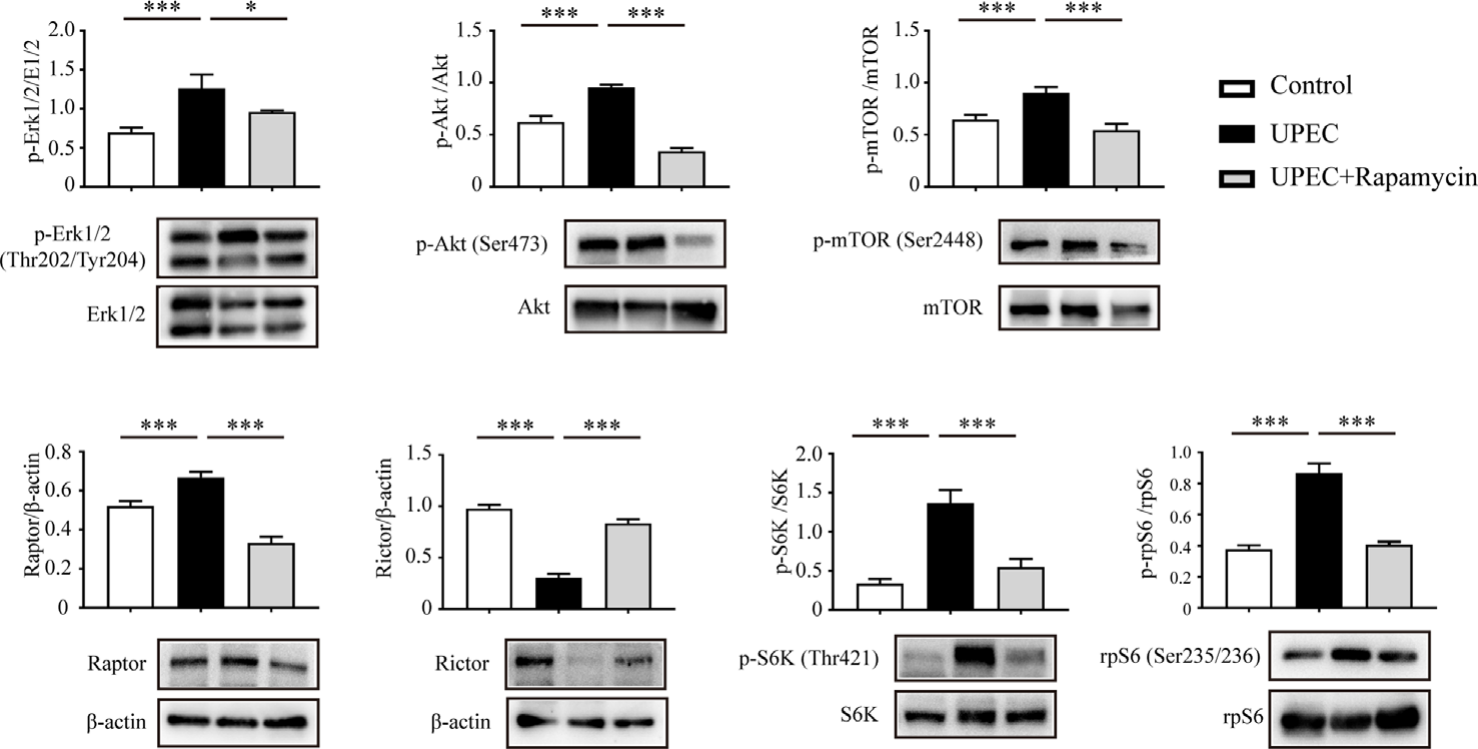
UPEC-derived soluble virulence factors activate the mTORC1-signaling pathway and disturb mTORC1-mTORC2 balance. Primary Sertoli cells were treated with UPEC culture supernatant for 40 hours, with either vehicle solution (UPEC) or with 0.1 μM rapamycin as supplement (UPEC + rapamycin). We extracted proteins in RIPA buffer and determined expression levels using western blotting analysis. Representative blots are shown. Data are presented as mean ± SD from triplicate experiments. Histograms show quantitative results, and *p* value was determined by ANOVA; *p < 0.05, ***p < 0.001.

**Figure 5:**
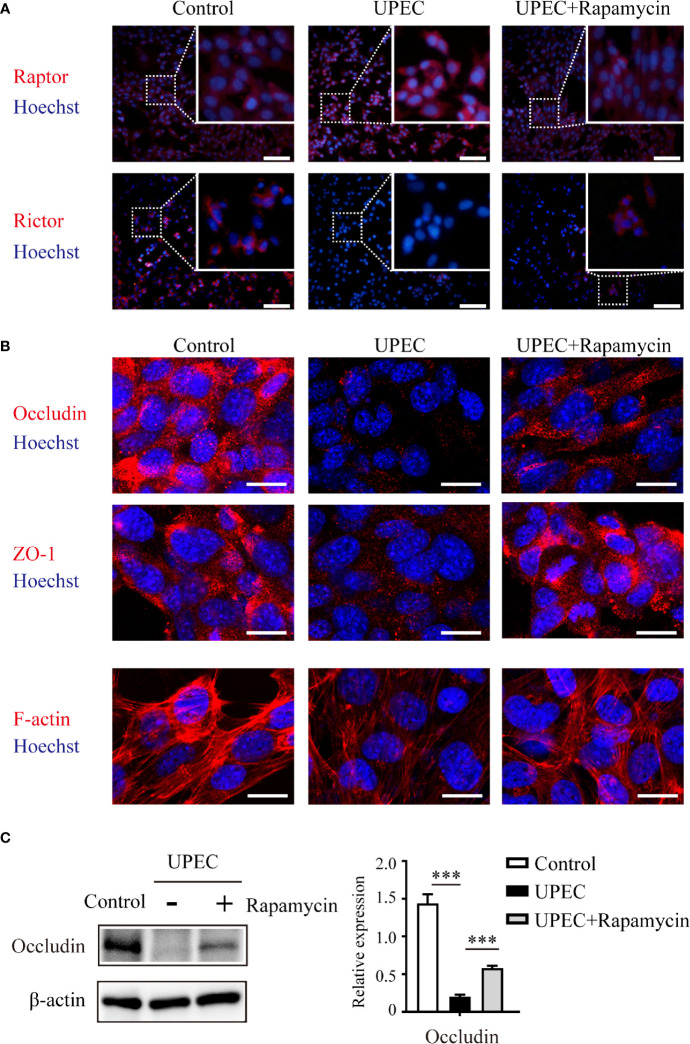
Rapamycin restores mTORC1-mTORC2 balance and rescues UPEC-induced down-regulation of cell-junction proteins in Sertoli cells. Primary Sertoli cells were treated with UPEC culture supernatant for 40 hours with vehicle solution (UPEC) or with 0.1 μM rapamycin supplement (UPEC+Rapamycin). Expression of Raptor and Rictor **(A)**, as well as cell-junction proteins **(B)**, was evaluated using immunofluorescence staining. Scale bars: **(A)** 50 μm, **(B)** 20 μm. **(C)** The occludin expression levels in Sertoli cells were further confirmed using western blotting, and representative blots are shown. Data are presented as mean ± SD from triplicate experiments. Histograms show quantitative results, and *p* value was determined by ANOVA; ***p < 0.001.

Our previous study indicated that the BTB appeared to remain functionally intact 7 days post-UPEC infection (21). However, the long-term effects of bacterial invasion on the BTB and the testicular immune microenvironment are still largely unknown. To further elucidate the possible changes of the BTB in orchitis-model rats over time, we first investigated alterations in BTB ultrastructure at different time-points within 1-month after infection (**Figure 1A**). In control rat testes (**Figure 1A-1**), tight junctions (TJ, indicated by white arrow), bundles of F-actin filaments (black arrowhead), and endoplasmic reticulum (ER, black arrow) in adjoining Sertoli cells were readily identified. There was no significant abnormality in TJs in the first few days after bacterial injection (**Figure 1A-2**), although actin filaments and ER became less distinct. As time passed, the structure of the TJs turned more obscure by Day 7 (**Figure 1A-3**) and even more indistinguishable at Days 14 and 30 (**Figures 1A-4, 5**). Moreover, the anti-sperm antibody showed positivity in infected animals three months after UPEC injection (**Figure 1B**). Collectively, our present findings provide a more complete observation on the dynamic alterations in BTB ultrastructure, suggesting that UPEC infection disturbed testicular immune privilege.

### The Expression of Sertoli Cell Junctional Proteins Decreases in the Testes of Orchitis-Model Rats

The BTB is composed of a series of junctional complexes between adjacent Sertoli cells—including tight junctions, gap junctions, basal ectoplasmic specialization (ES, a testis-specific actin-rich adhesion junction), and desmosomes. To further investigate the impact of UPEC infection on the BTB, we evaluated the expression patterns of Sertoli cell junctional proteins at different time-points post infection using immunofluorescence staining (**Figure 2**). Occludin, one of the tight-junction proteins, was observed between neighboring Sertoli cells and germ cells in control testes. However, occludin expression was significantly decreased from day 3 after infection, continually declined at later time-points, and was barely detectable in affected seminiferous tubules on day 14. A similar time-dependent pattern of decreasing expression was found for tight junction adaptor protein zonula occludens-1 (ZO-1), the cell-skeleton protein filamentous actin (F-actin), gap junction protein connexin-43 (CX-43), and adhesion junction proteins (β-catenin and N-cadherin). These results confirmed that UPEC infection induced extensive down-regulation of a variety of BTB constituent proteins in the testis of rats induced with orchitis.

### UPEC Bacterial Supernatant Disrupts Connections Between Sertoli cells and Down-regulates the Expression of Cell Junctional Proteins

To verify the influence of UPEC infection on the connections between Sertoli cells, we treated primary Sertoli cells and the Sertoli cell line TM4 with UPEC culture supernatant (**Figure 3**). Although adjacent TM4 cells were observed to be closely attached to each other in the control group using SEM, cells became more and more dissociated after bacterial supernatant stimulation in a dose- and time-dependent manner (**Figure 3A**). In an *in vitro* culture system of primary Sertoli cells, we evaluated the expression of occludin, ZO-1, and F-actin. Similar to what we observed in orchitis-model rat testes, the expression of these 2 cell-junction proteins was markedly diminished in primary Sertoli cells treated with a low dose of UPEC supernatant for 40 hours (**Figures 3B, C**); the down-regulation of junctional proteins was even more significant when the dose was increased. The density of the F-actin network (which was stained with CoraLite^®^594-conjugated phalloidin) was found to be negligible and disorganized in infected primary Sertoli cells. Collectively, our findings using *in vivo* and *in vitro* models provided compelling evidence that indicated that UPEC infection decreased Sertoli cell junctional protein expression and eventually undermined the BTB.

### mTORC1-mTORC2 Balance Is Disturbed in Infected Sertoli Cells

Recent studies suggest that the balance between mTORC1 and mTORC2 plays a pivotal role in the regulation of BTB dynamics (13, 26). We thus investigated the impact of UPEC infection on the mTOR-signaling pathway using an *in vitro* model. In primary Sertoli cells treated with UPEC bacterial culture supernatant, we observed increased phosphorylation of both Erk1/2 and Akt (**Figure 4**), which are the upstream enzymes regulating the mTORC1 pathway (27, 28). Consequently, we found mTORC1 activation as indicated by the phosphorylation of mTOR Ser2448, as well as the upregulation of Raptor, whereas the key component of mTORC2—Rictor—was accordingly downregulated by approximately 70% in Sertoli cells treated with bacterial culture supernatant. P70 ribosomal S6 kinase (S6K)—the downstream substrate of mTORC1—was subsequently activated and, consequently, the phosphorylation of the S6K substrate—S6 ribosomal protein (rpS6)—was also significantly increased. Significantly, each of the changes observed in the investigated signaling pathways was abolished by rapamycin treatment. These findings suggest that mTORC1 was over-activated while mTORC2 was suppressed in Sertoli cells during UPEC infection.

### Rapamycin Partially Rescues the Down-regulation of Cell Junctional Proteins in Infected Sertoli Cells

Considering the role of mTOR signaling on BTB regulation, we next evaluated the possibility of restoring the mTORC1-mTORC2 balance and the expression of junctional proteins in Sertoli cells using the mTORC1-specific inhibitor rapamycin (**Figure 5**). Consistent with the above findings using immunoblotting, it was confirmed in infected Sertoli cells *via* immunofluorescence that Raptor and Rictor were upregulated and downregulated, respectively. Of note, these impacts were abolished by administering a rapamycin supplement (**Figure 5A**) that induced the suppression of mTORC1 and reactivation of mTORC2. Importantly, the administration of rapamycin partially restored the expression and distribution of cell- junction proteins that we observed in primary Sertoli cells (**Figures 5B, C**). These findings further suggested an mTORC1-mTORC2 imbalance may contribute to the disruption of Sertoli cell connections caused by UPEC infection.

### The Expression of Sertoli Cell Junctional Proteins Is Restored by Rapamycin in Orchitis-Model Rats

**Figure 6:**
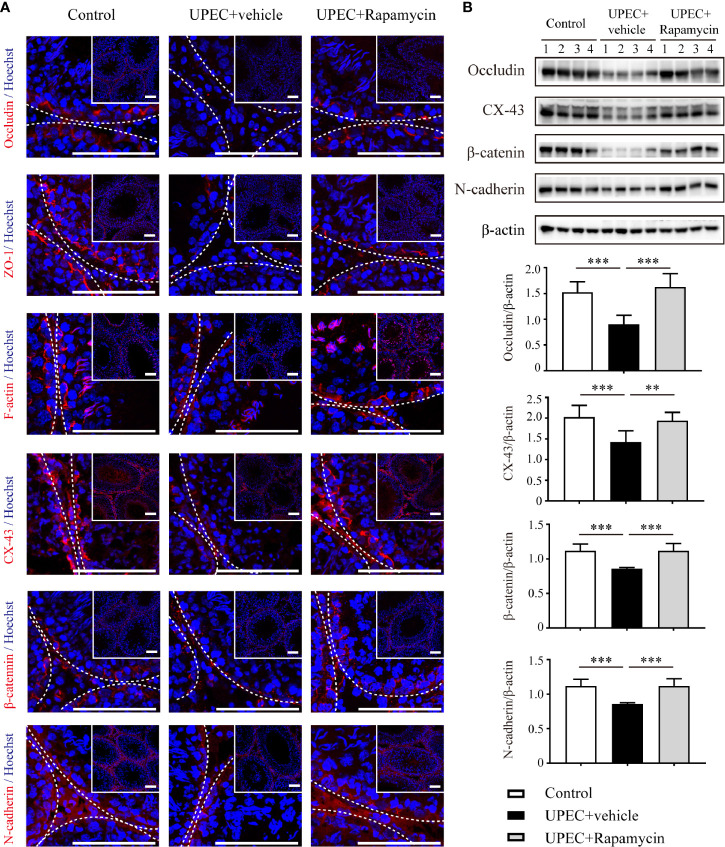
Rapamycin (5 mg/kg/day) treatment for seven days partially rescues down-regulation of cell-junction proteins in UPEC-infected testes. **(A)** We evaluated cell-junction protein expression in all three groups using immunofluorescence staining and confocal microscopy. The images in the insets are the same testes cross-sections at lower magnification (scale bar, 100 μm). The white broken lines indicate the relative location of the basement membrane adjacent to the BTB of the seminiferous tubules. **(B)** Representative blots show the expression patterns of selected junctional proteins in the testes from all three groups animals (n=4 in each group). Histograms show quantitative results, and *p* value was determined by ANOVA; **p < 0.01, ***p < 0.001.

To assess the *in vivo* effectiveness of rapamycin treatment on restoring Sertoli cell junctional proteins, we administrated rapamycin (5 mg/kg/day) daily from day 7 onward for one extra week in UPEC-infected rats. Animals were sacrificed on day 14 and sections of rat testes were collected (as described in Materials and Methods). We determined the expression of a series of cell-junction proteins on frozen sections of testes using immunofluorescence (**Figure 6A**) and, similar to the results observed through *in vitro* experiments, each of these proteins were down-regulated by approximately 30–50% in UPEC-infected rats. Notably, the expression of selected cell-junction proteins rebounded moderately after one week of rapamycin treatment. Similar results were further confirmed by immunoblotting analysis using total testicular tissue proteins (**Figure 6B**). These data confirmed that rapamycin partially restored the expression of BTB proteins after UPEC infection.

### Rapamycin Treatment Partially Reinforces BTB Integrity in Infected Rats Without Additional Spermatogenic Disruption

**Figure 7:**
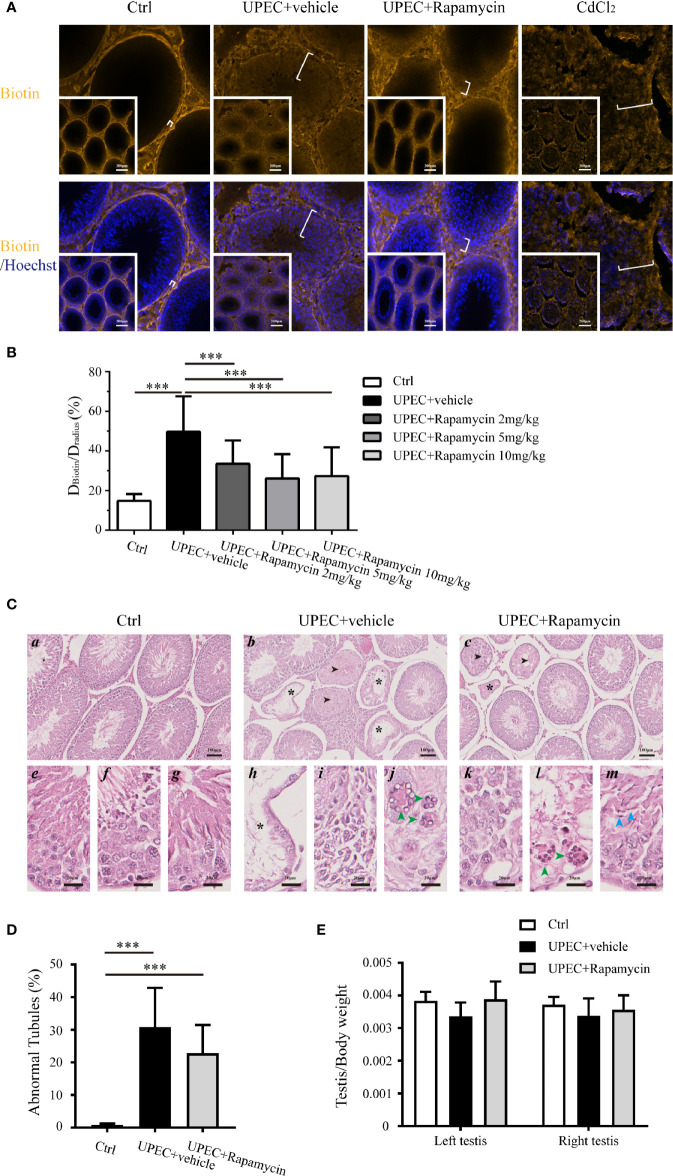
Rapamycin treatment reduces biotin infiltration through the BTB and does not perturb spermatogenesis in testes from UPEC-infected rats. **(A)** BTB integrity was functionally evaluated using a sulfo-NHS-LC-biotin diffusion assay, and the representative images of biotin diffusion in the testes of different groups are shown. The testes of CdCl_2_-treated rats served as positive controls. The white brackets indicate the distance penetrated by biotin. The images in the insets are the same testes cross sections at lower magnification (scale bar, 200 μm). **(B)** Semi-quantified data from the sulfo-NHS-LC-biotin diffusion assay are demonstrated as a bar graph. For an oval-shaped tubule from an oblique cross-section, we calculated the radius using the mean of the longest and shortest radii. Approximately 50 tubules were randomly scored per testis. D_Biotin_, distance penetrated by biotin in seminiferous tubule; D_radius_, the radius of the corresponding seminiferous tubule. *p* value was determined with ANOVA, ***p<0.001. **(C)** Paraffin sections of testes (4 μm) were stained with hematoxylin and eosin. Histopathologic evaluation was performed on the testes of control (n=4), UPEC + vehicle-treated (n=6), and rapamycin-treated animals (n= 6) using light microscopy. The images were captured using a NanoZoomer S210 system and representative figures are depicted (*a*-*c*: scale bar, 100 μm; *e*-*m*: scale bar, 20 μm). Seminiferous tubules marked with asterisks indicate Sertoli cell-only tubules (*b*, *c*, *h*); those marked with arrowheads indicate hypospermatogenic tubules (*b and c*). *e–g*: higher-magnification images showing seminiferous epithelium at stages II-III, VIII, and XIII in control rat testes, respectively; *h*–*j*: higher-magnification images demonstrating typical pathologic changes in UPEC-infected rats testes (*h*, extensive germ-cell loss, and only Sertoli cells remained in the seminiferous epithelium; *I*, infiltration of inflammatory cells in the interstitial compartment; *j*, the presence of multinucleated, round spermatids are indicated by green arrowheads); *k*–*m*, higher-magnification images show pathologic changes in testes from orchitis-model animals treated with rapamycin (*k*, disordered seminiferous epithelium with germ cell exfoliation; *l*, the presence of multinucleated round spermatids are indicated by green arrowheads; *m*, elongated spermatids with defects in polarity are indicated by blue arrowheads, in which the spermatid heads were misoriented by pointing 45°–90° away from the basement membrane). **(D)** The percentage of abnormal seminiferous tubules in each testicular cross-section was calculated by assessing over 200 tubules in every sample. The data are presented as mean ± SD and shown in the histogram. *p* value was determined by ANOVA; ***p<0.001. **(E)** The ratios of testis to body weight in the three groups are presented as mean ± SD and shown in the histogram. *p* value was determined using ANOVA.

To further investigate the potential value of rapamycin treatment in BTB maintenance in rats with orchitis, BTB integrity was evaluated using a biotin-infiltration assessment (**Figures 7A, B**). The testes of CdCl_2-_treated rats served as positive controls since this chemical is known to cause irreversible disruption of the BTB (29). In the saline-injected control group, the BTB remained intact as biotin migration was restricted to the basement membrane instead of penetrating into the abluminal compartment. In contrast, the BTB was completely compromised in the testes of CdCl_2_-treated animals as indicated by the infiltration of biotin into the lumen of the seminiferous tubules. Similarly, BTB integrity was also greatly abolished in UPEC-infected rat testes, with biotin freely penetrating across the abluminal compartment in a majority of tubules. Intriguingly, the distance of biotin infiltration was significantly reduced in rapamycin-treated orchitis rats, supporting the assumption that rapamycin contributed to the maintenance of BTB integrity in orchitis.

It has been reported, however, that long-term administration of rapamycin leads to a reversable impairment of spermatogenesis by inhibiting mTOR signaling (30–32). Given the function of mTORC1, it is reasonable to question whether 7-day treatment with rapamycin further perturbs spermatogenesis in orchitis animals. Therefore, a histopathological assessment was performed on the cross-sections of testes (**Figures 7C, D**), and the ratios of testis to body weights in three (control, UPEC-infected and vehicle-treated, and UPEC-infected and rapamycin-treated) groups were calculated (**Figure 7E**). Consistent with our previous report (21), UPEC infection caused various degrees of spermatogenic impairment and immune cell infiltration (**Figures 7C** *b*, *h*–*j*). Importantly, the number of abnormal seminiferous tubules was slightly lower in the rapamycin-treated group (22.7 ± 8.6%) compared with vehicle-treated animals (30.8 ± 15%), although this difference was not statistically significant (**Figure 7D**). Furthermore, the ratios of testis to body weights were similar in control and rapamycin groups on day 14, whereas the ratios in UPEC-infected and vehicle-treated groups showed a tendency to be lower (although again not statistically significant) (**Figure 7E**). Taken together, our findings suggest a role for short-term rapamycin treatment in BTB protection, without inducing further spermatogenic dysfunction during UPEC infection.

## Discussion

We previously demonstrated that the BTB appeared to remain functionally intact at one single time-point in the early stage of UPEC infection (21). In addition, we also observed that the bacteria migrated across the seminiferous epithelium and reached the interstitial compartment of the testis (21). We therefore speculated that UPEC either utilized epithelial transcytosis or caused a transient disruption of the BTB to reach the interstitium. Considering the role of the BTB in the maintenance of testicular immuno-privilege, the impacts of infection or inflammation on this barrier have attracted increasing attention. Wu et al. found that the mumps virus could disrupt the BTB through the induction of TNF-α in Sertoli cells (19), and other investigators reported that a lipopolysaccharide (LPS) challenge induced occludin down-regulation and compromised BTB integrity (33). In our previous study we demonstrated UPEC-derived α-hemolysin caused Sertoli cell necrosis by inducing calcium influx and mitochondrial dysfunction (34). Given that UPEC bacteria possess multiple virulence factors that include LPS and α-hemolysin, it is reasonable to assume that the presence of UPEC inside the testis would disrupt the BTB formed by neighboring Sertoli cells. Herein, we therefore investigated the continuous dynamic changes in the BTB during UPEC invasion, and partially deduced the potential mechanism underlying BTB impairment in orchitis.

Compared to the infection models we had used previously, we slightly modified the UPEC-infection model used in the present study. For the *in vivo* orchitis model, a lower number of bacteria were directly injected into the testis rather than through the vas deferens, as pathogens can migrate to the interstitium (21). In addition, the UPEC culture supernatant was applied to *in vitro* experimentation primarily based upon two rationales. First, our previous studies indicated that soluble virulence factors, such as α-hemolysin, are the dominant factors inducing Sertoli cell necrosis (34), and second, in our experience MOI (multiplicity of infection) is difficult to control precisely in cell-culture experiments over several days due to the constant proliferation of bacteria. With our modified infection models, we first demonstrated that UPEC invasion caused structural and functional impairment of the BTB in a time-dependent manner. Moreover, our results suggested that an mTORC1-mTORC2 imbalance plays a pivotal role in BTB disruption, which can be partially restored by the specific mTORC1 inhibitor rapamycin.

Rather than only examining samples on day 7 post-infection as in our previously published research, in the present study we observed ultrastructural disruption of the BTB at multiple time-points using TEM, which showed additional and significant morphologic changes over time. We also found anti-sperm antibody positivity in the sera of orchitis-model rats after three months of modeling. Using *in vivo* and *in vitro* models, our results showed that UPEC infection or bacterial culture supernatant decreased to varying degrees of the expression of Sertoli cell junctional proteins that comprise the BTB. These findings confirmed a progressive destruction of the BTB by bacterial invasion, and subsequent compromise of the testicular immune-privileged microenvironment.

A series of experiments have shown that BTB cell-junction proteins are not only essential for barrier integrity, but also indispensable for supporting spermatogenesis. For example, occludin-knockout mice were infertile by 36–60 weeks of age, with an absence of spermatocytes and spermatids in the seminiferous tubules (35, 36). It was also reported that spermatogonia failed to differentiate beyond type A in CX-43 Sertoli cell specific-knockout mice (37). Moreover, BTB assembly delay during postnatal development caused by diethylstilbestrol treatment resulted in meiotic failure of spermatocytes and a delay in spermiation (38). Indeed, we also noted spermatogenic dysfunction in our UPEC-induced orchitic animals that was accompanied by BTB destruction. Given our previously published findings, the down-regulation of BTB cell-junction proteins in response to UPEC infection may be an important mechanism contributing to degeneration of the seminiferous epithelium in our orchitis model. In this context, safeguarding BTB integrity in orchitis is particularly important in preserving testicular function.

The underlying molecular mechanisms controlling BTB “opening” and “closing” are of particular interest in the context of orchitis. A number of recent studies indicated that the delicate balance between mTORC1 and mTORC2 occupies a key role in the regulation of BTB restructuring under normal physiologic conditions (13, 14). However, the role of mTORC1-mTORC2 in orchitis-related BTB disruption remains unclear. Herein, we noted that mTORC1, as well as the upstream kinases Erk1/2 and Akt, were all activated, whereas mTORC2 was suppressed in Sertoli cells facing a challenge from UPEC-soluble virulence factors. The hyperactivation of mTORC1 and attenuation in mTORC2 both promote an “open/leaky” state with respect to the BTB, and this imbalance likely contributes to the disruption of barrier integrity during UPEC infection. We also observed that rapamycin, an mTORC1- specific inhibitor, abolished mTORC1 hyperactivation and partially restored the expression of all our selected junctional proteins in infected Sertoli cells. Consistent with our *in vitro* data, we also confirmed the rescue effects of rapamycin in an orchitis rat model following UPEC infection. This further suggested that disequilibrated mTORC1-mTORC2 contributed to UPEC-induced BTB damage, which could then provide a potential therapeutic target in orchitis.

Previous studies have shown that mTOR signaling is directly involved in the nutritional support of spermatogenesis (39). In addition, mTOR is required for the proliferation and differentiation of spermatogonial stem cells as opposed to survival and maintenance (40). Serra et al. established that raptor-knockout led to incomplete meiosis by spermatogonia and no production of mature spermatozoa (41). Busada et al. demonstrated that mTORC1 inhibition with rapamycin resulted in a blockade of spermatogonial differentiation (42). Infertility is consistently one of the most common side effects experienced by men undergoing organ transplantation and long-term rapamycin treatment to prevent organ rejection (31, 43, 44). This immunosuppressant potentially causes a reversible diminution in a series of sperm parameters such as sperm count, motility, and vitality (32, 45). Given the critical role of mTORC1 in germ cell differentiation and rapamycin’s side effects on fertility, it is of critical importance to ascertain whether short-term rapamycin treatment is detrimental to spermatogenesis. Congruent with our earlier study, UPEC infection induced considerable disruption of spermatogenesis and induced the infiltration of immune cells. It is noteworthy that we uncovered herein a tendency to decrease in the number of abnormal tubules in infected rats treated with rapamycin compared to the vehicle-treated controls (although this was not statistically significant). This suggests that at least seven days of rapamycin treatment (up to 10 mg/kg) at the acute phase of infection may actually exert a protective effect instead of aggravating the degeneration of seminiferous epithelium in infected testes. Thus, this may constitute a promising finding that sheds new light on the treatment of orchitis, providing an alternative to the sole use of antibiotics.

However, there are still some limitations to the present study. For example, it will be necessary in the future to fully evaluate the safety of our remedy strategy using rapamycin alone in control animals, and to investigate the impact on spermatozoa. Further study is also required to explore the long-term effects on spermatogenesis and fertility. It was recently reported that a rapamycin analog with 40 times more selectivity for mTORC1 reduced off-target inhibition of mTORC2, substantially decreasing metabolic and immunologic side effects (46). However, further investigation is required to determine the optimal dosage and treatment duration with an appropriate mTORC1 inhibitor. Moreover, it is essential to identify the principal virulence factor(s) that induces BTB dysfunction to better understand the underlying mechanism(s) of orchitis-related infertility.

In summary, the present study illustrated that an imbalance in mTORC1-mTORC2 contributed to disruptive alterations of the BTB following UPEC infection. Restoring these two complexes to their normal antagonistic function may provide a complementary strategy for treating bacterial orchitis. Our results thus revealed the possibility of further protecting testicular structure and function from infection/inflammation-induced tissue damage.

## Data Availability Statement

The raw data supporting the conclusions of this article will be made available by the authors, without undue reservation.

## Ethics Statement

The animal study was reviewed and approved by the Ethical Committee of the Zhongshan Hospital.

## Author Contributions

NY and YL designed the experiments, YL wrote the manuscript, and NT provided manuscript revision. YL, ML, QC, and BY performed the cell culture and molecular biology experiments. YL and ML performed the animal experiments and analyzed the data, and XC and XD provided intellectual input and coordination. All authors contributed to the article and approved the submitted version.

## Funding

This work was funded by project grants from the National Natural Science Foundation of China (nos. 81300473, 82071643, and 81971345). 

## Conflict of Interest

The authors declare that the research was conducted in the absence of any commercial or financial relationships that could be construed as a potential conflict of interest.

## Supplementary Material

Supplementary Table 1 List of antibodies.

Supplementary Figure 1 Immunofluorescence staining of vimentin in primary Sertoli cells Supplementary PowerPoint Original western blots.

Supplementary PowerPoint Original western blots.
