# Supplementary figures and images for: Uropathogenic Escherichia coli Infection Compromises the Blood-Testis Barrier by Disturbing mTORC1-mTORC2 Balance
Source: Front Immunol. 2021 Feb 19;12:582858. doi: 10.3389/fimmu.2021.582858 (PMC7933507; doi:10.3389/fimmu.2021.582858)

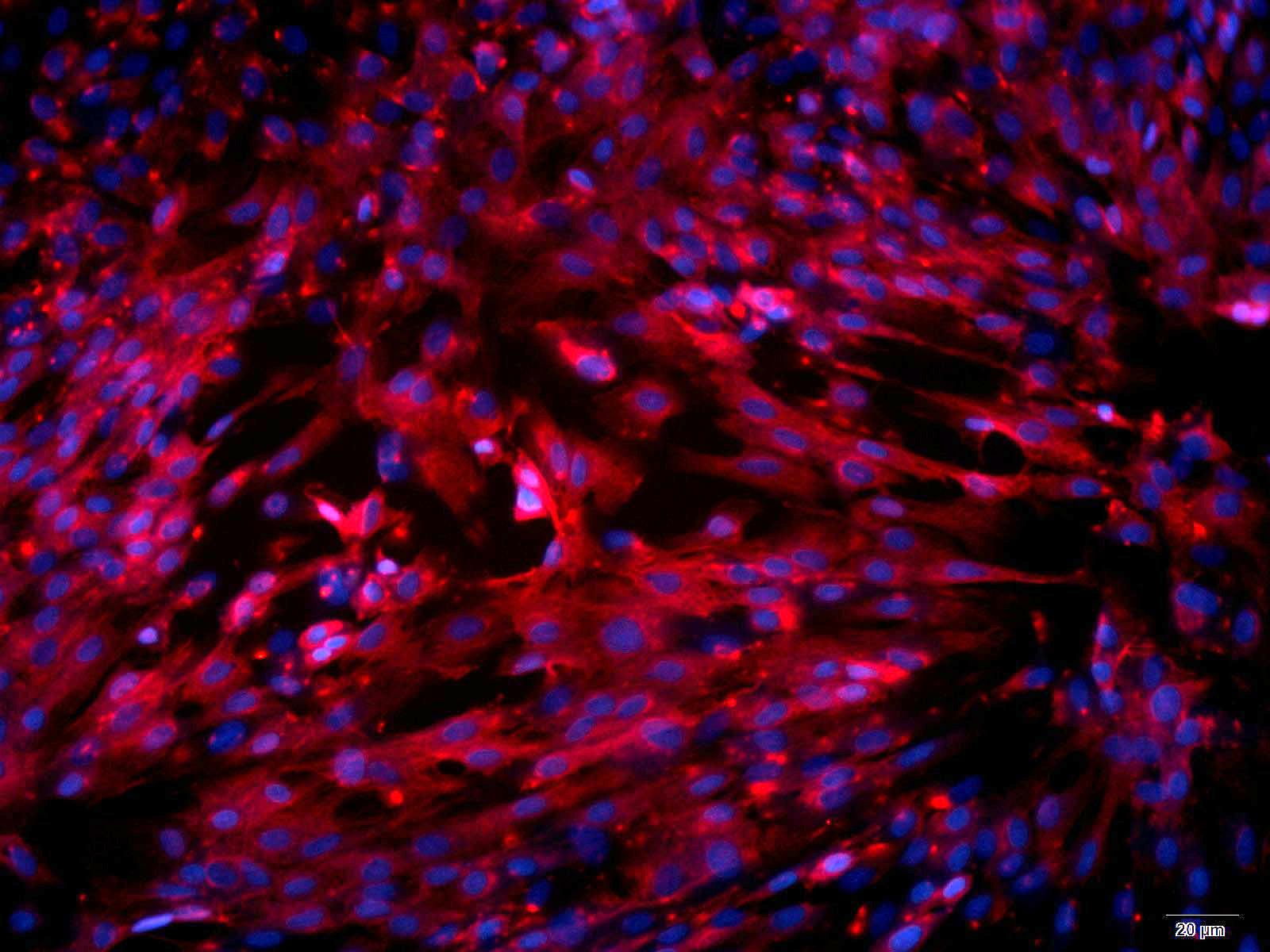

Supplement: Supplementary Figure 1 — Immunofluorescence staining of vimentin in primary Sertoli cells Supplementary PowerPoint Original western blots. [file Image_1.tif]
