# Supplementary material for: Uropathogenic Escherichia coli Infection Compromises the Blood-Testis Barrier by Disturbing mTORC1-mTORC2 Balance
Source: Front Immunol. 2021 Feb 19;12:582858. doi: 10.3389/fimmu.2021.582858 (PMC7933507; doi:10.3389/fimmu.2021.582858)
Supplement: Supplementary PowerPoint — Original western blots. [file Presentation_1.ppt]

## Slide 1
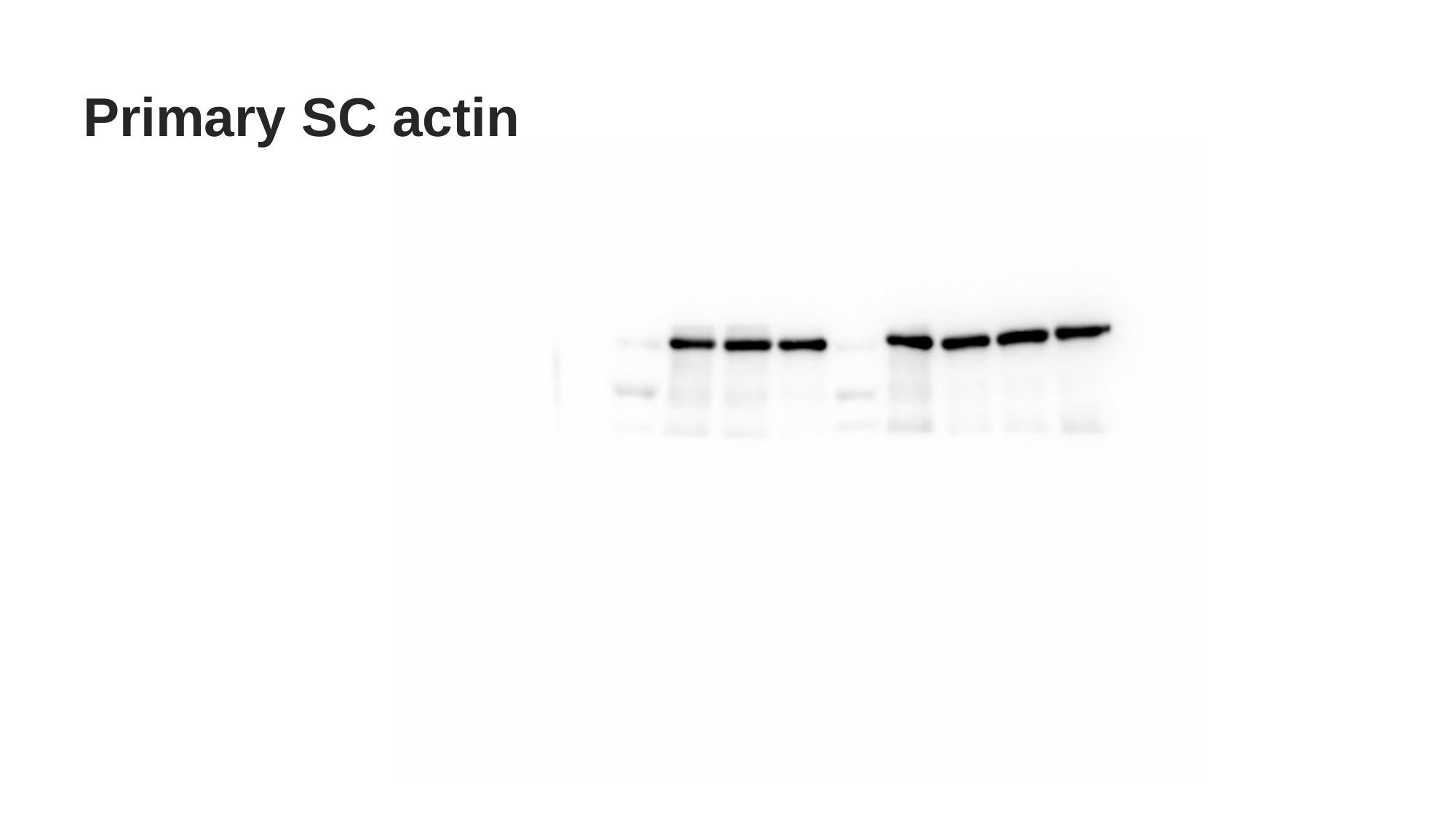

# Primary SC actin

## Slide 2
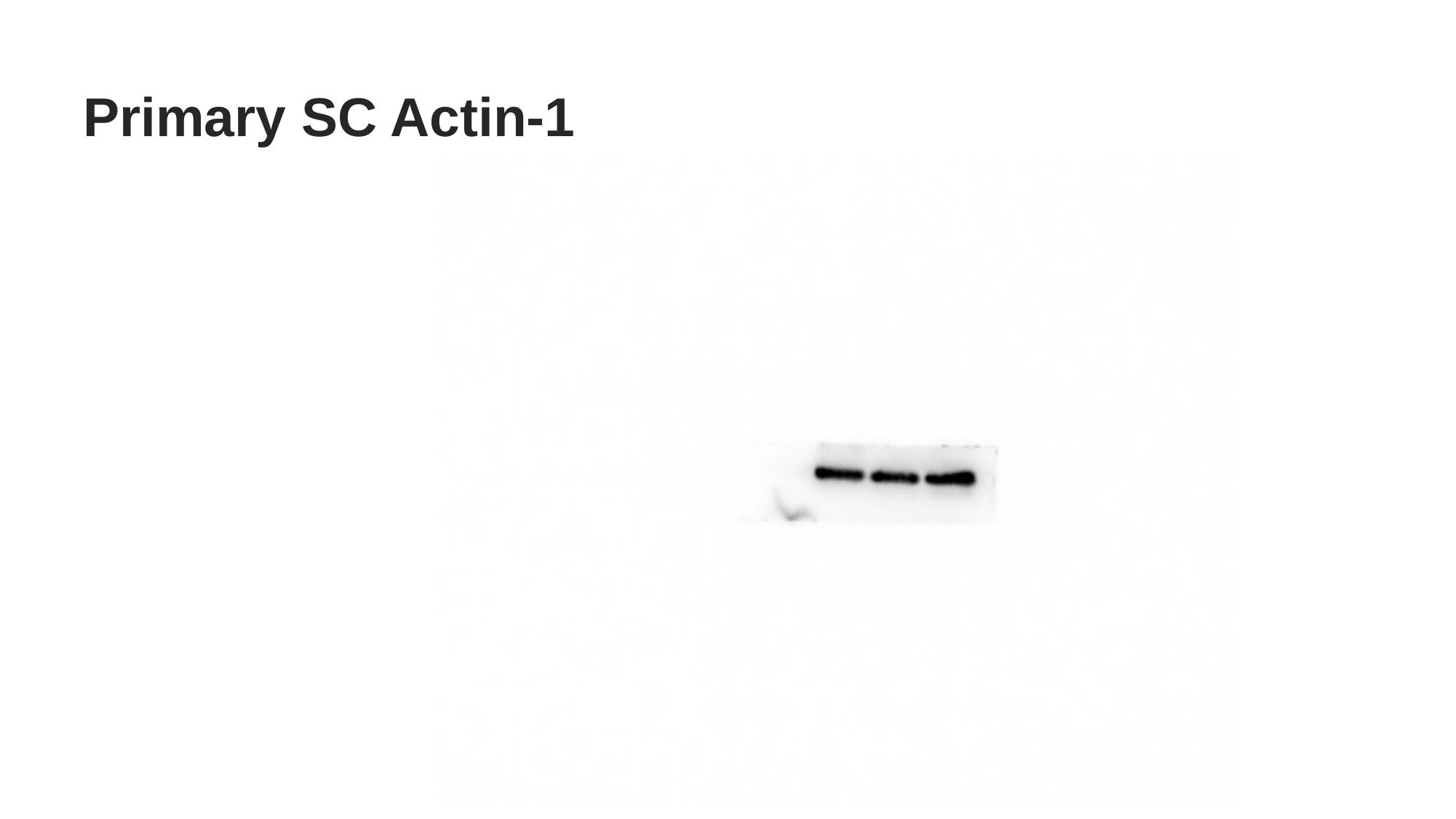

# Primary SC Actin-1

## Slide 3
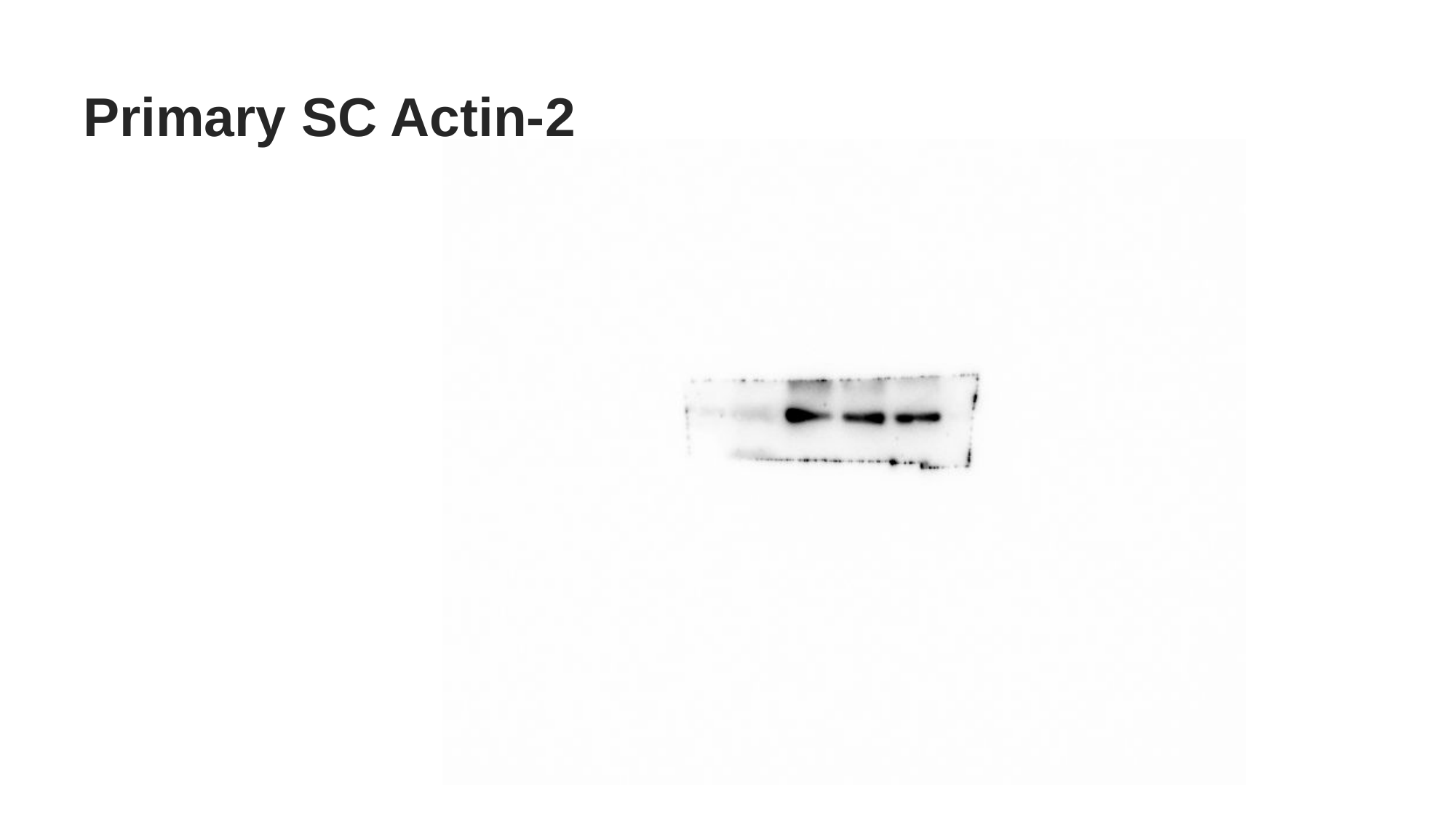

# Primary SC Actin-2

## Slide 4
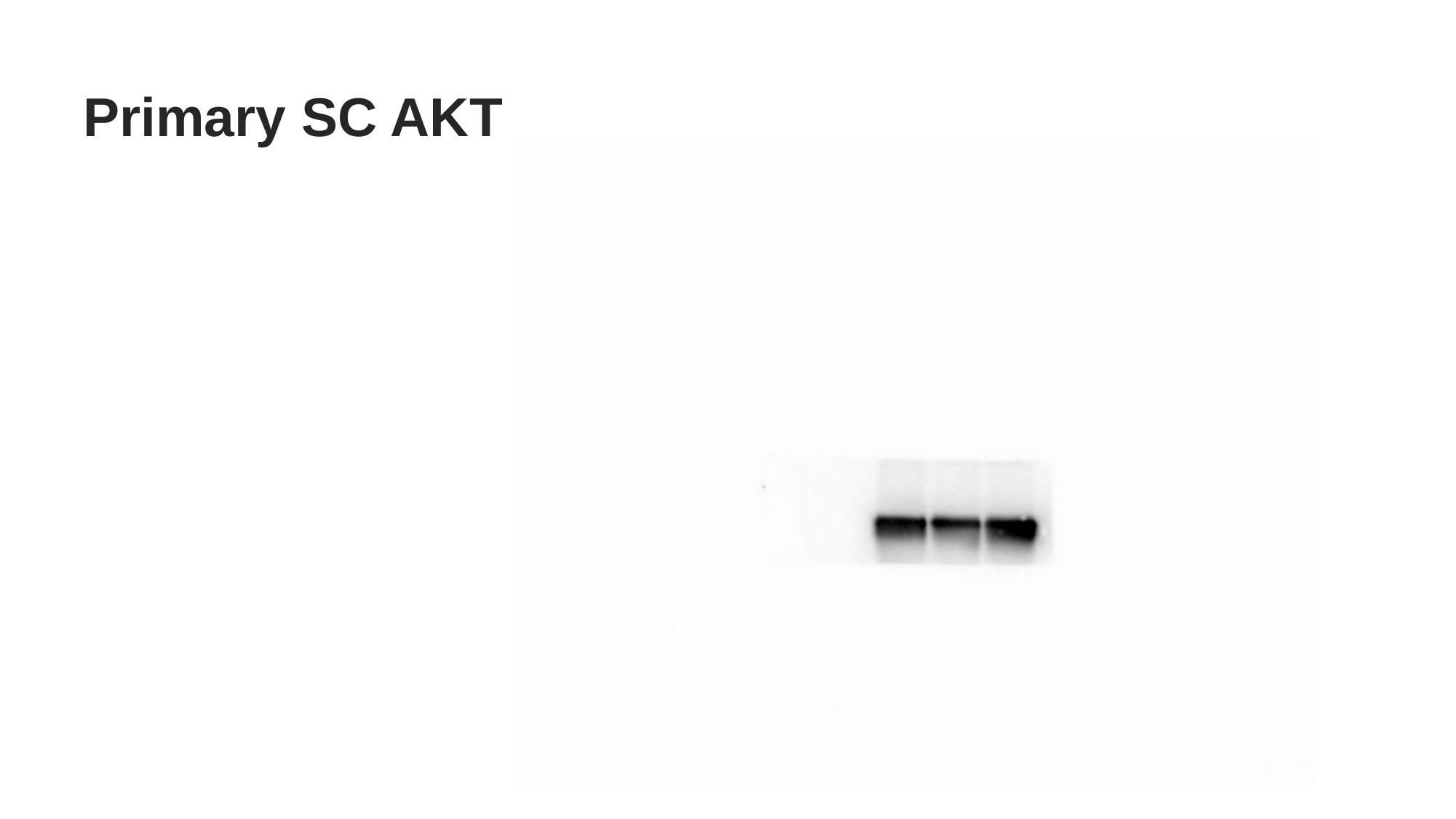

# Primary SC AKT

## Slide 5
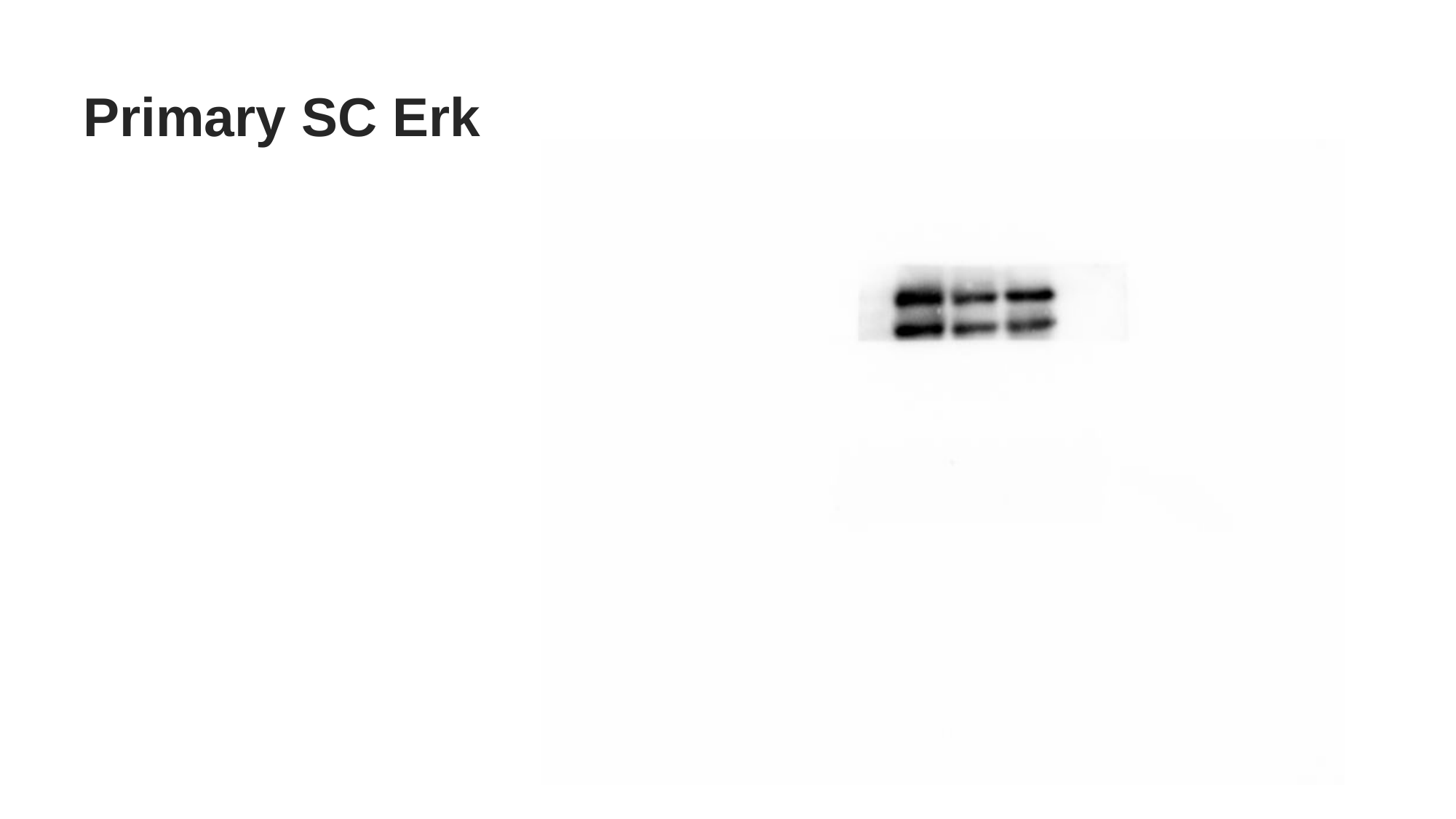

# Primary SC Erk

## Slide 6
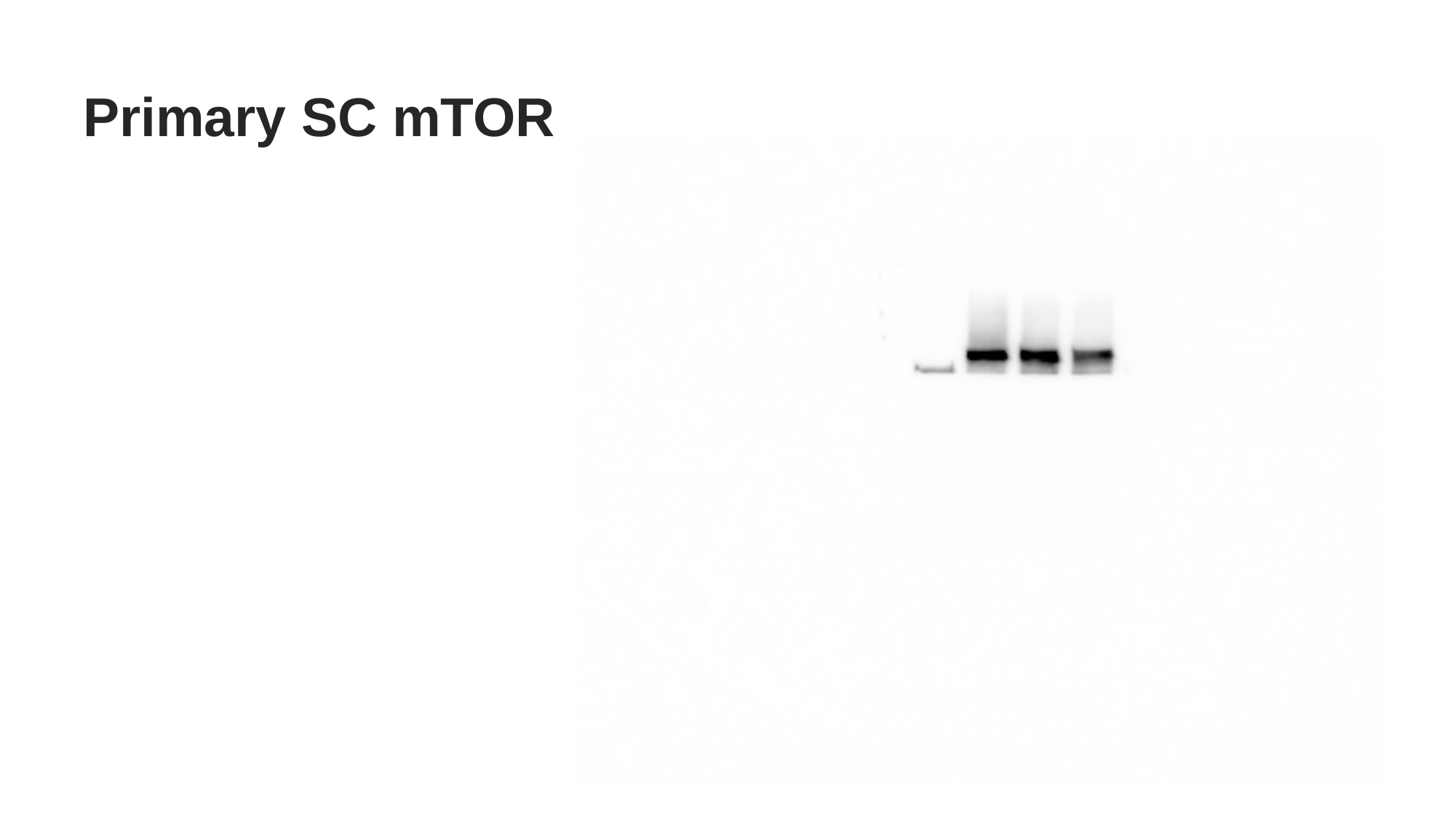

# Primary SC mTOR

## Slide 7
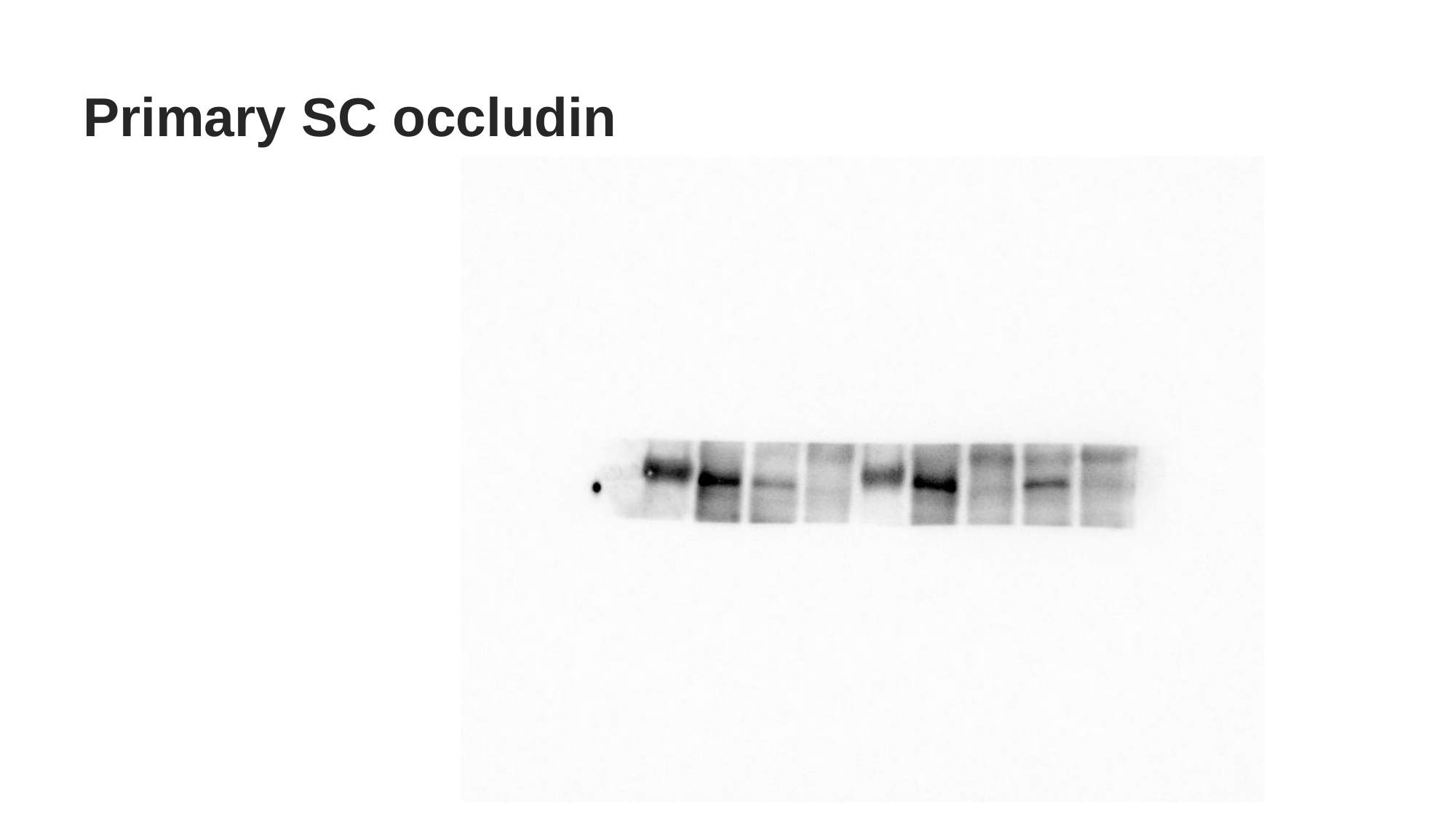

# Primary SC occludin

## Slide 8
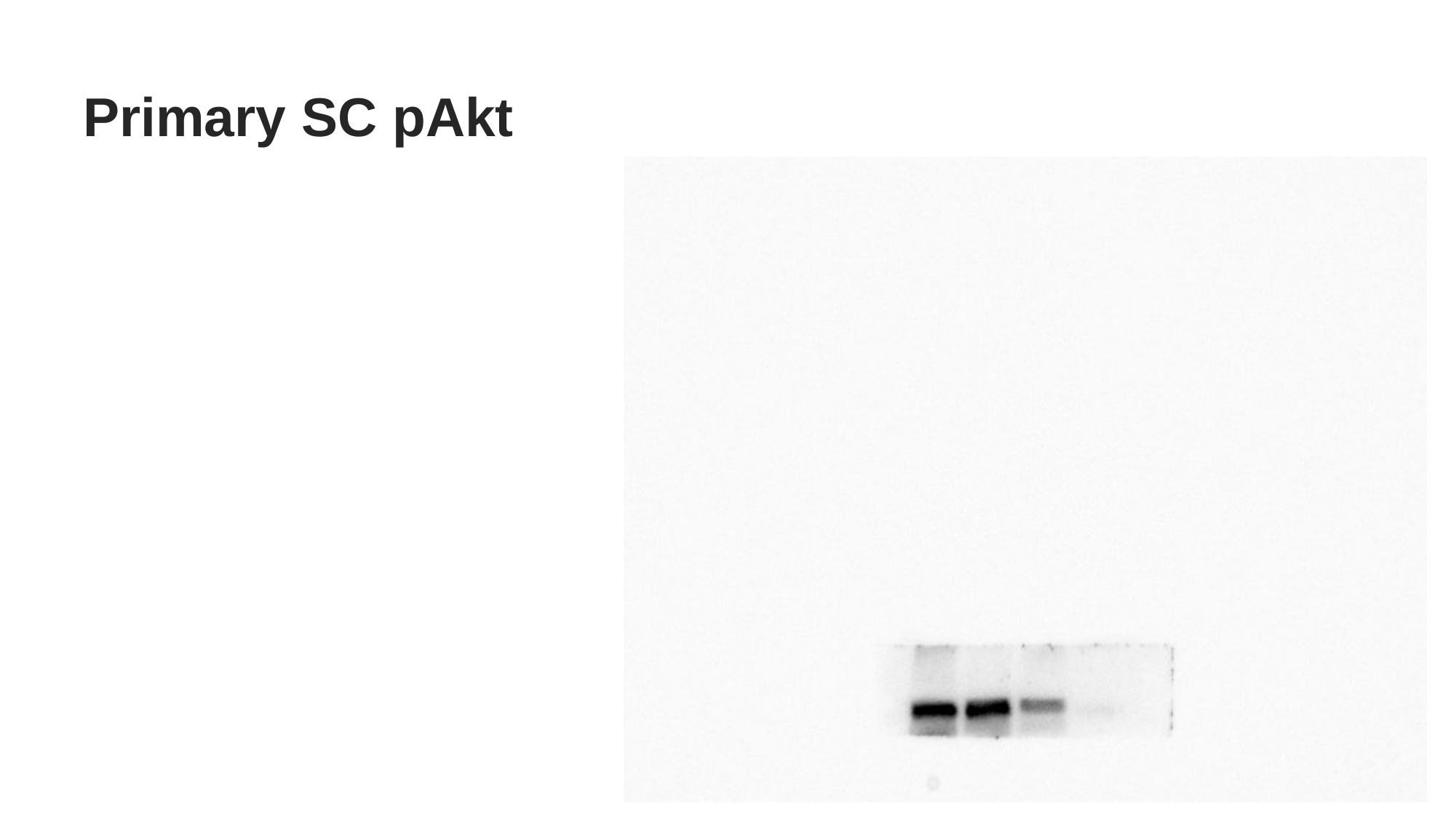

# Primary SC pAkt

## Slide 9
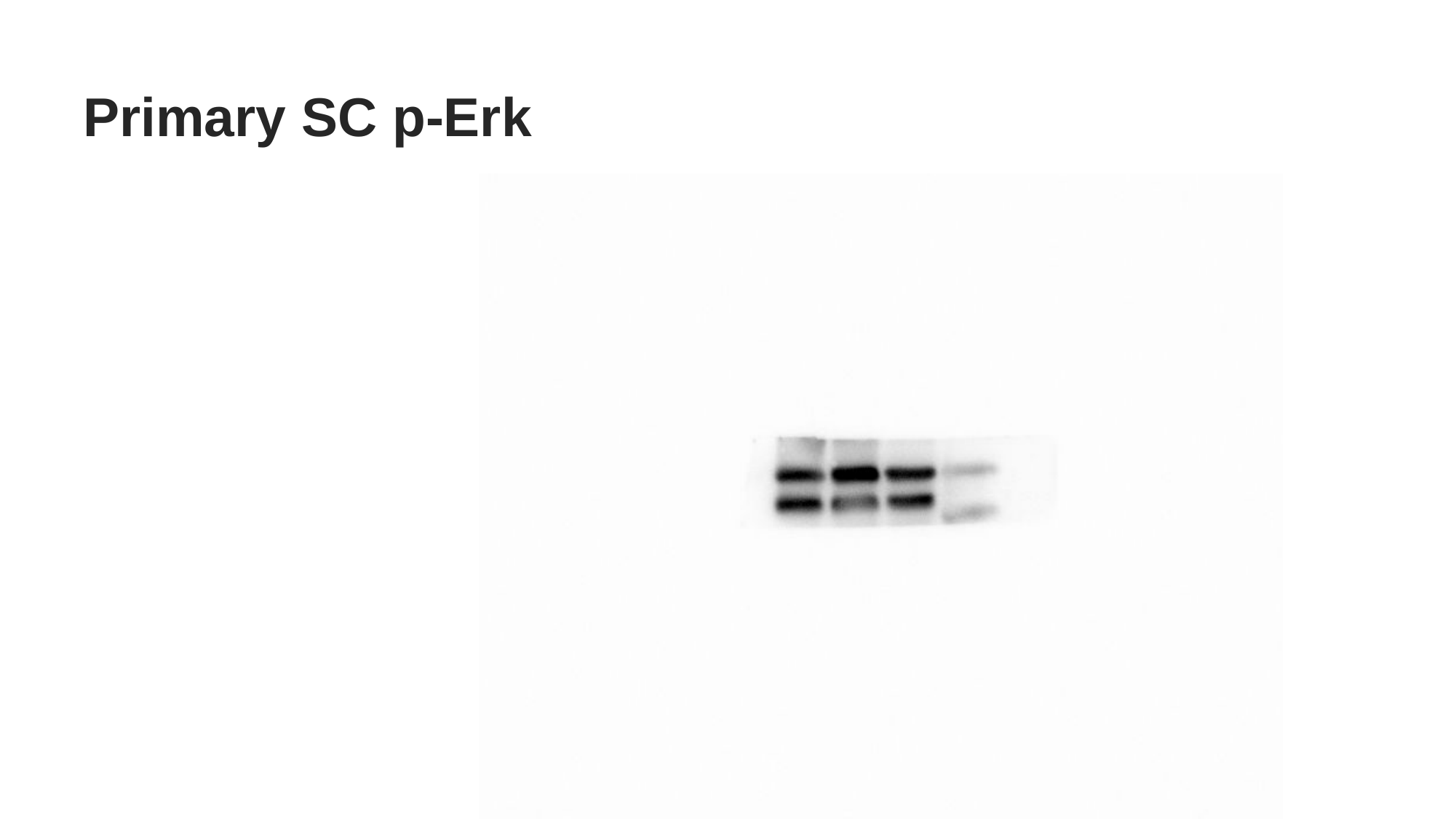

# Primary SC p-Erk

## Slide 10
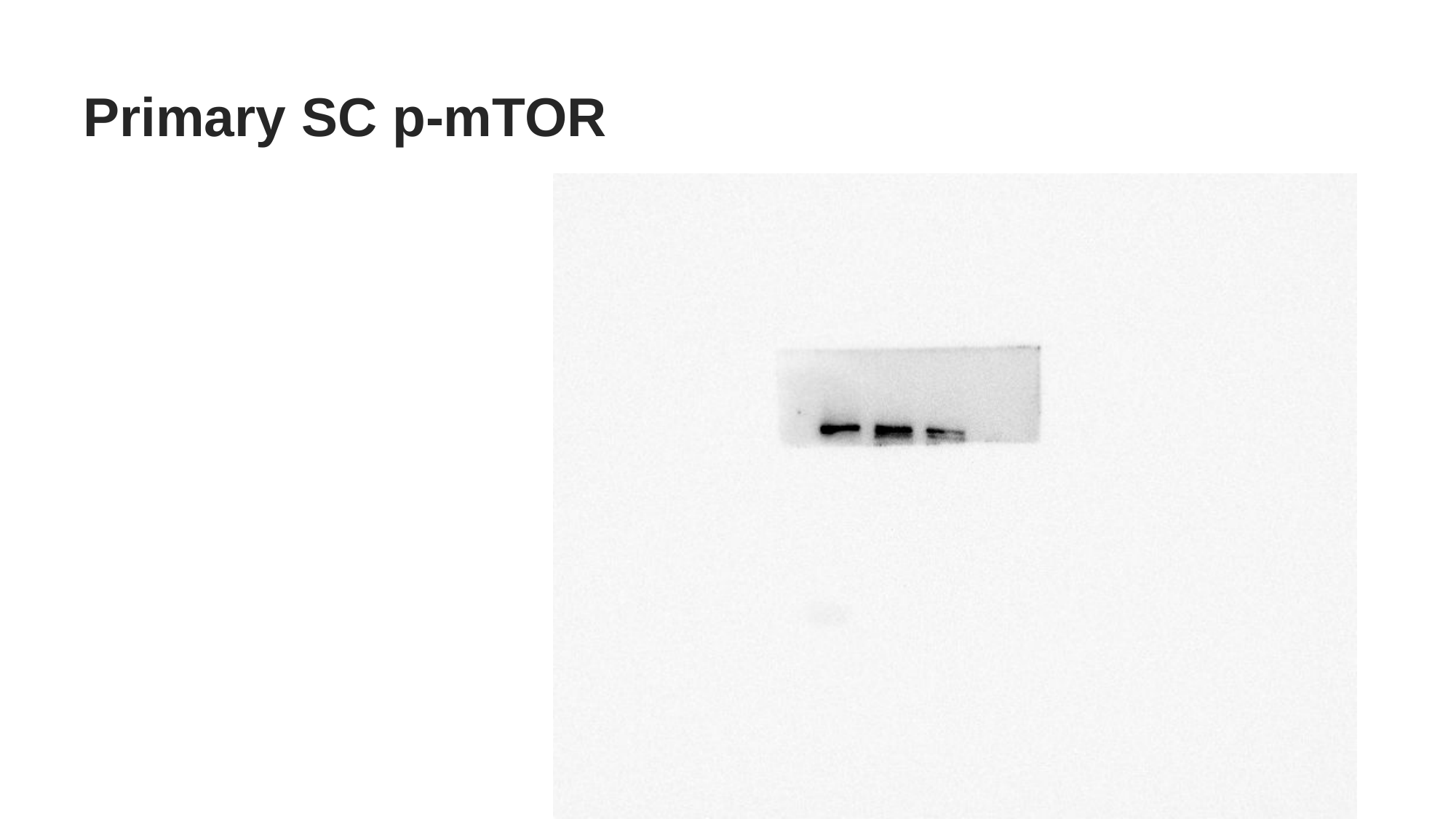

# Primary SC p-mTOR

## Slide 11
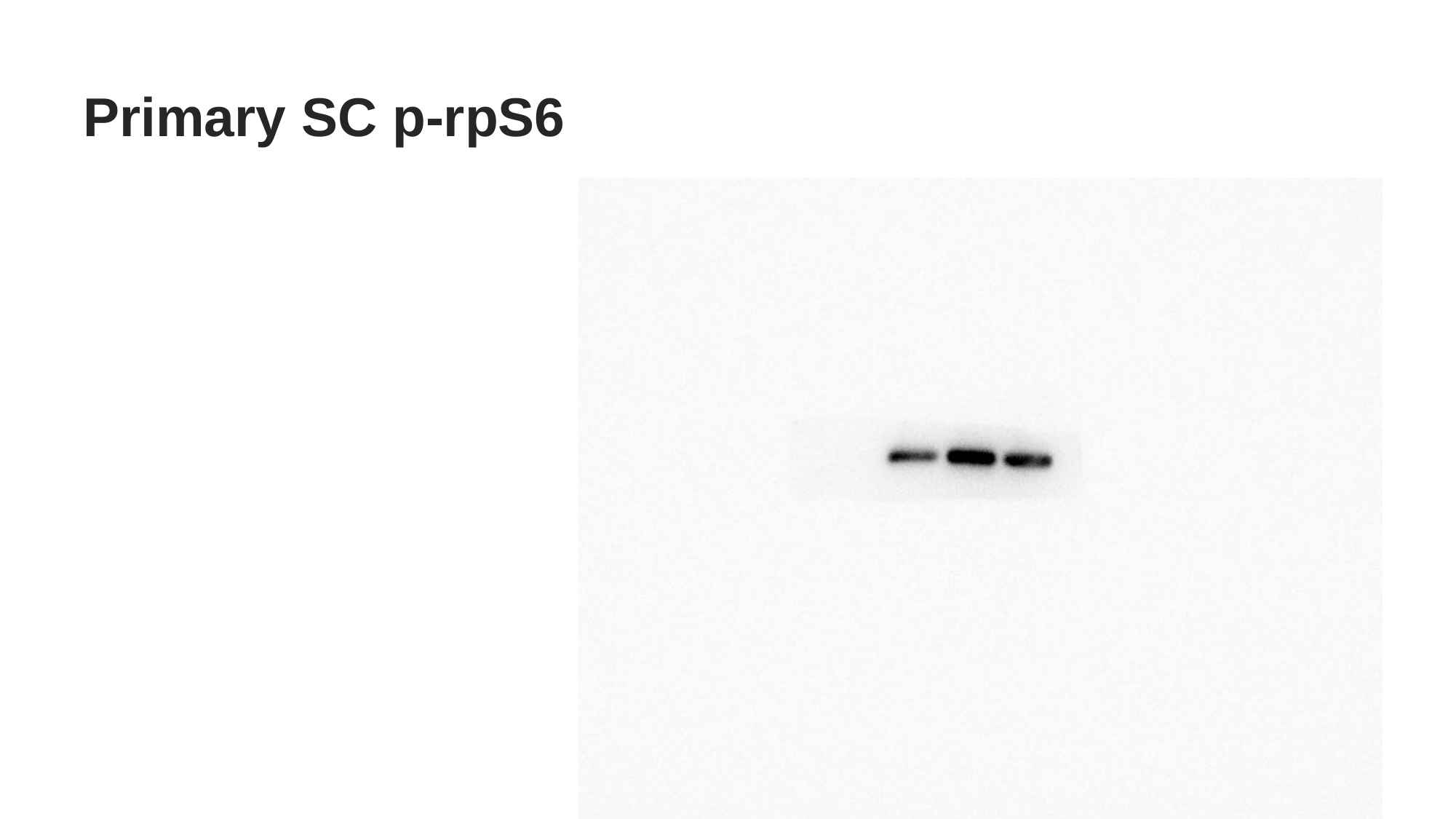

# Primary SC p-rpS6

## Slide 12
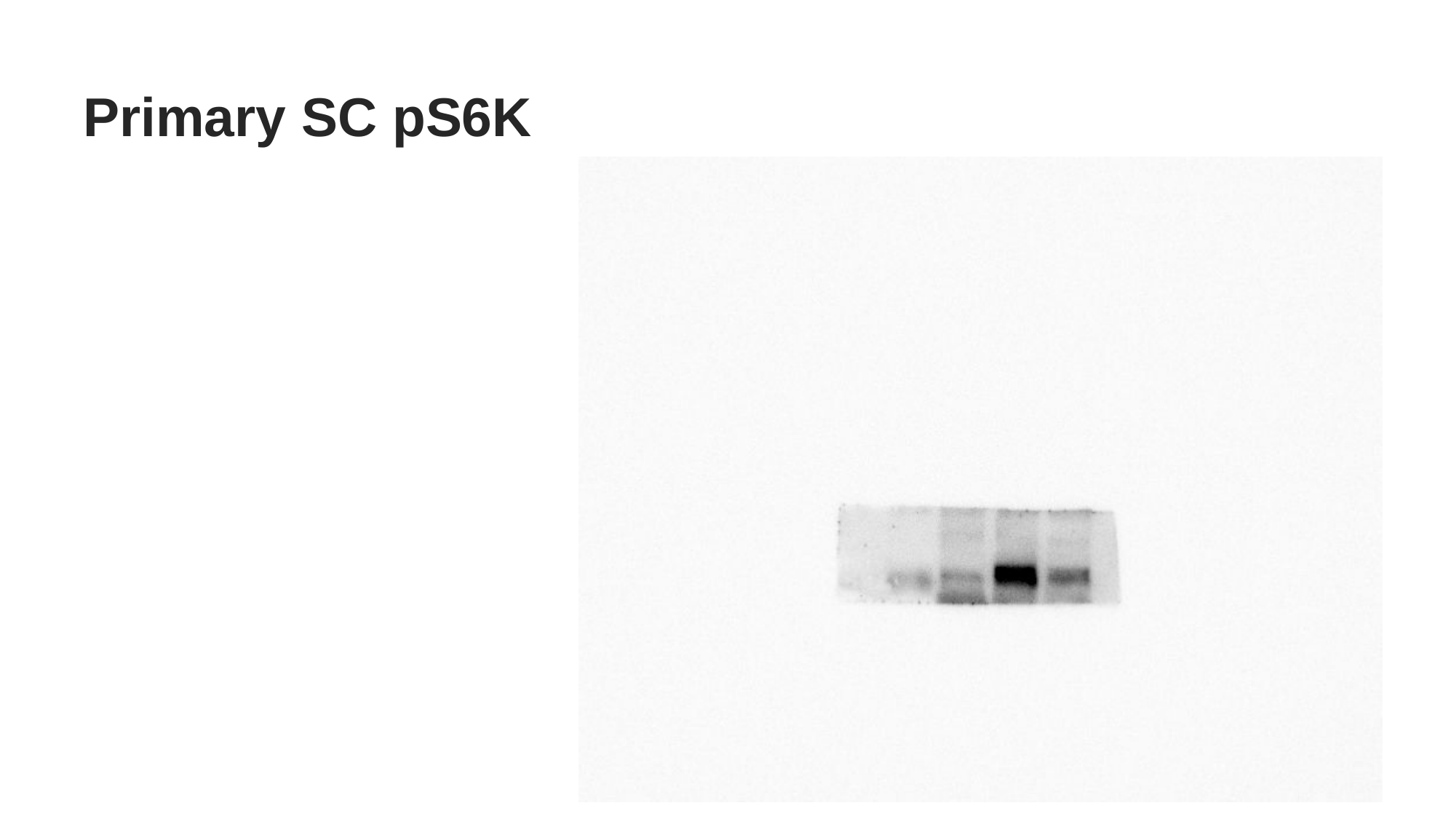

# Primary SC pS6K

## Slide 13
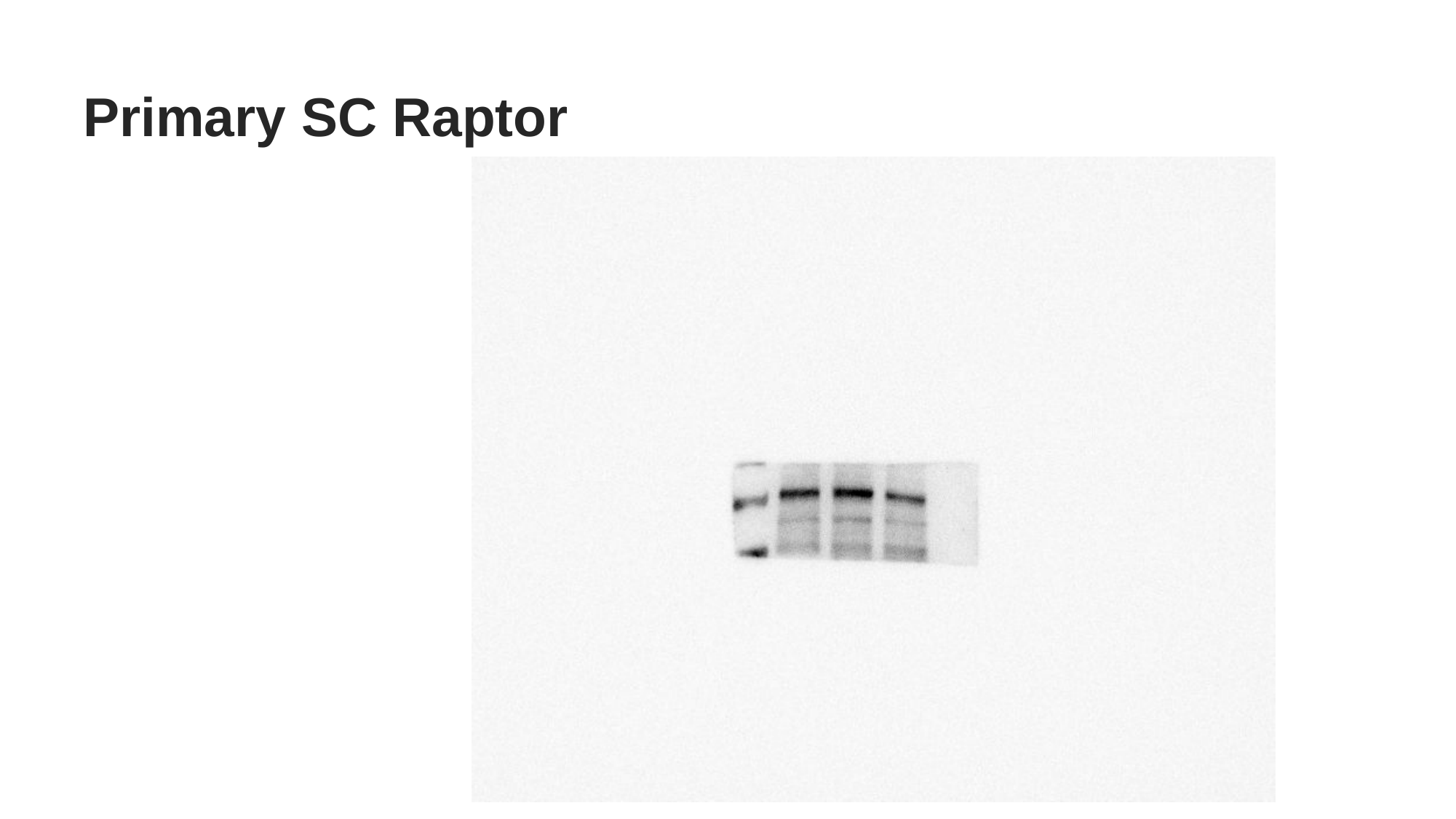

# Primary SC Raptor

## Slide 14
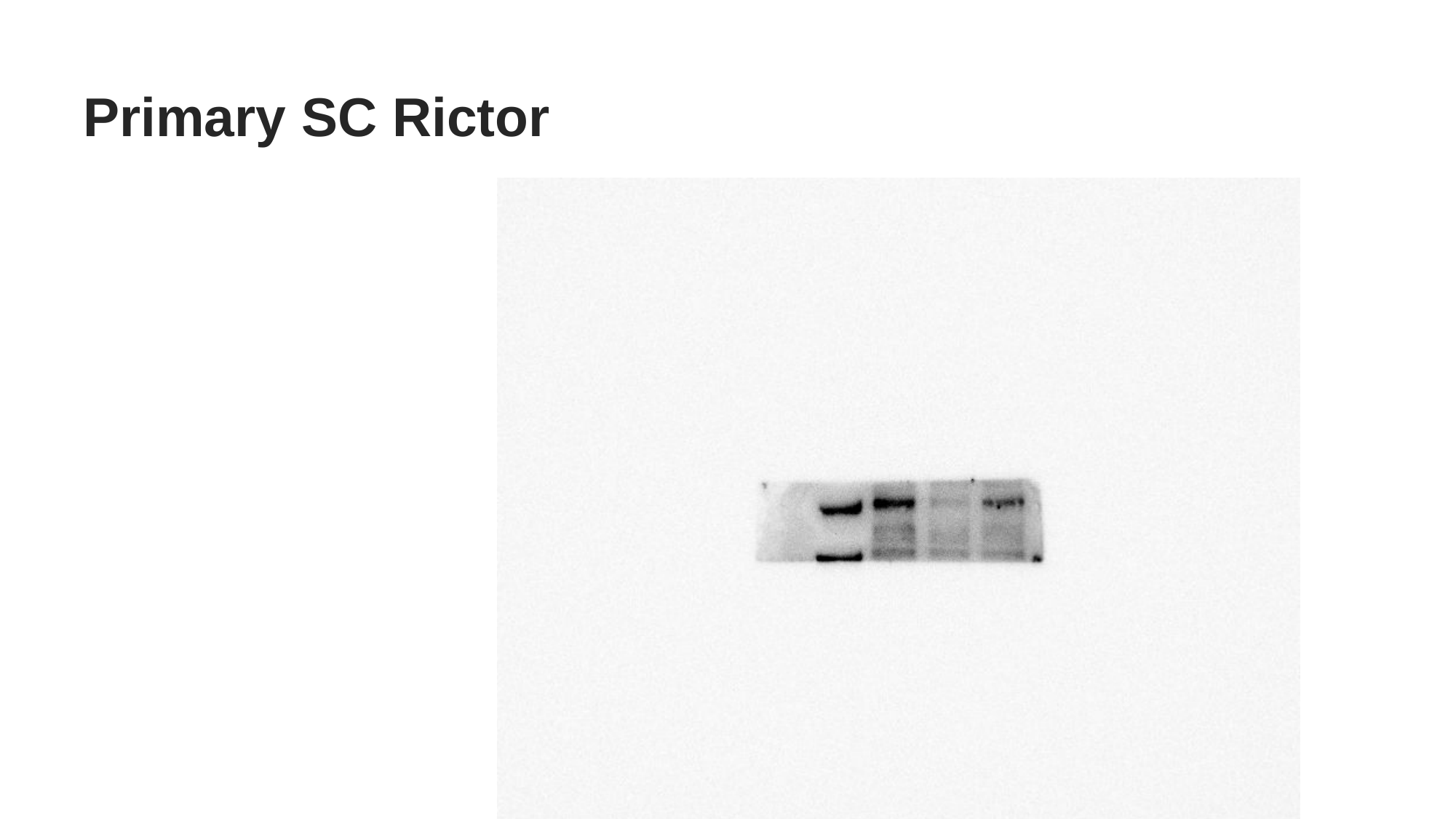

# Primary SC Rictor

## Slide 15
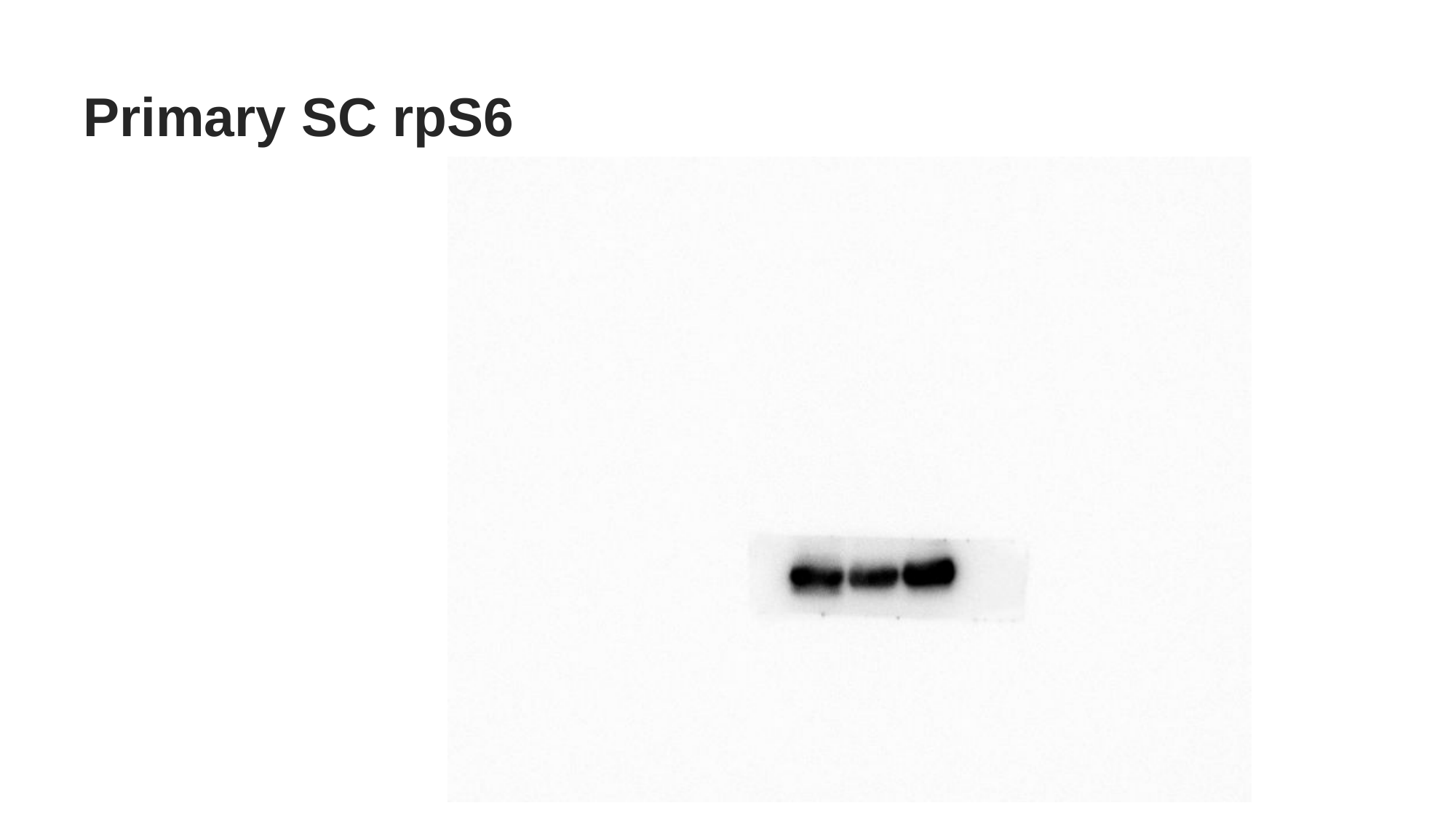

# Primary SC rpS6

## Slide 16
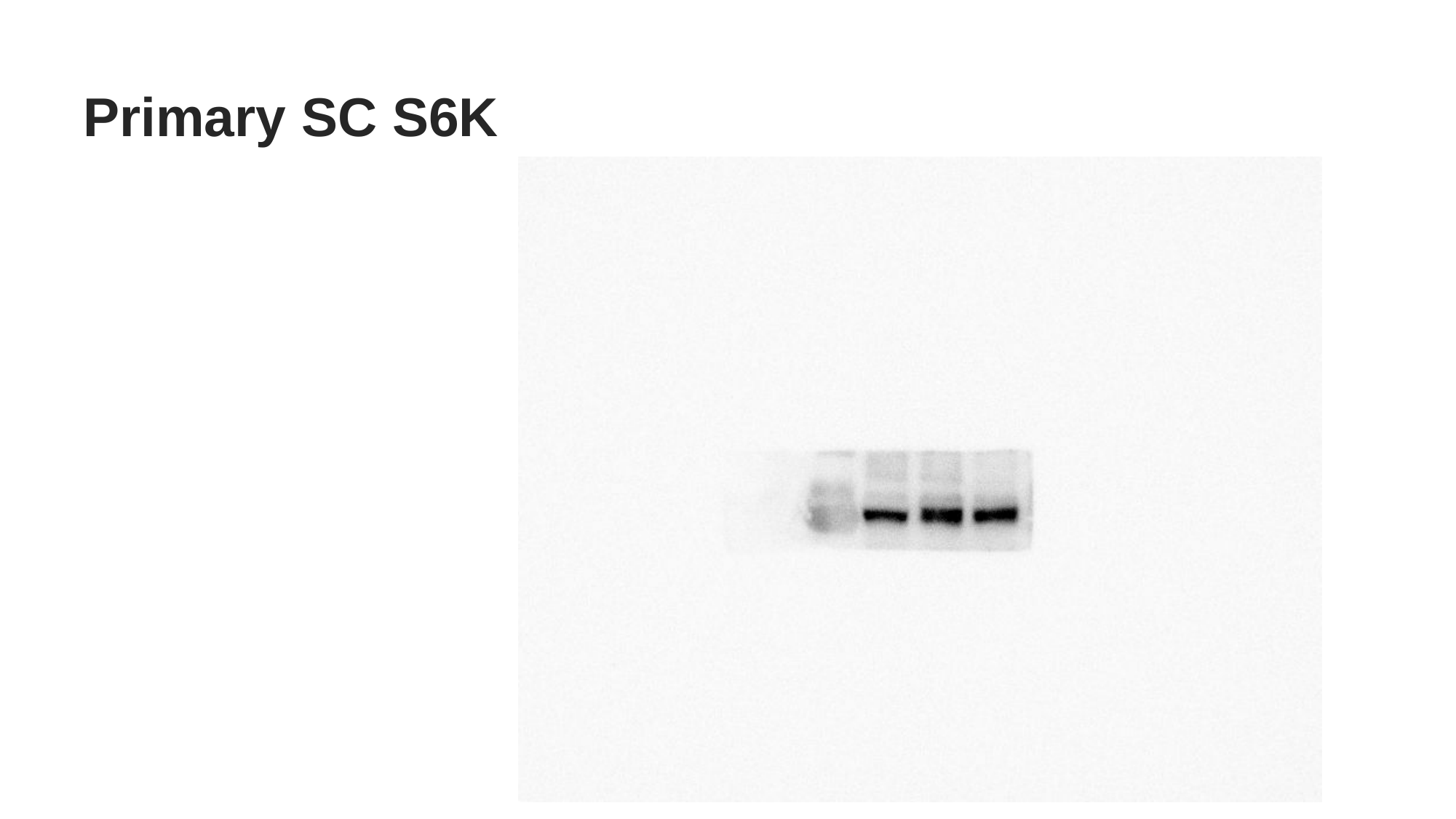

# Primary SC S6K

## Slide 17
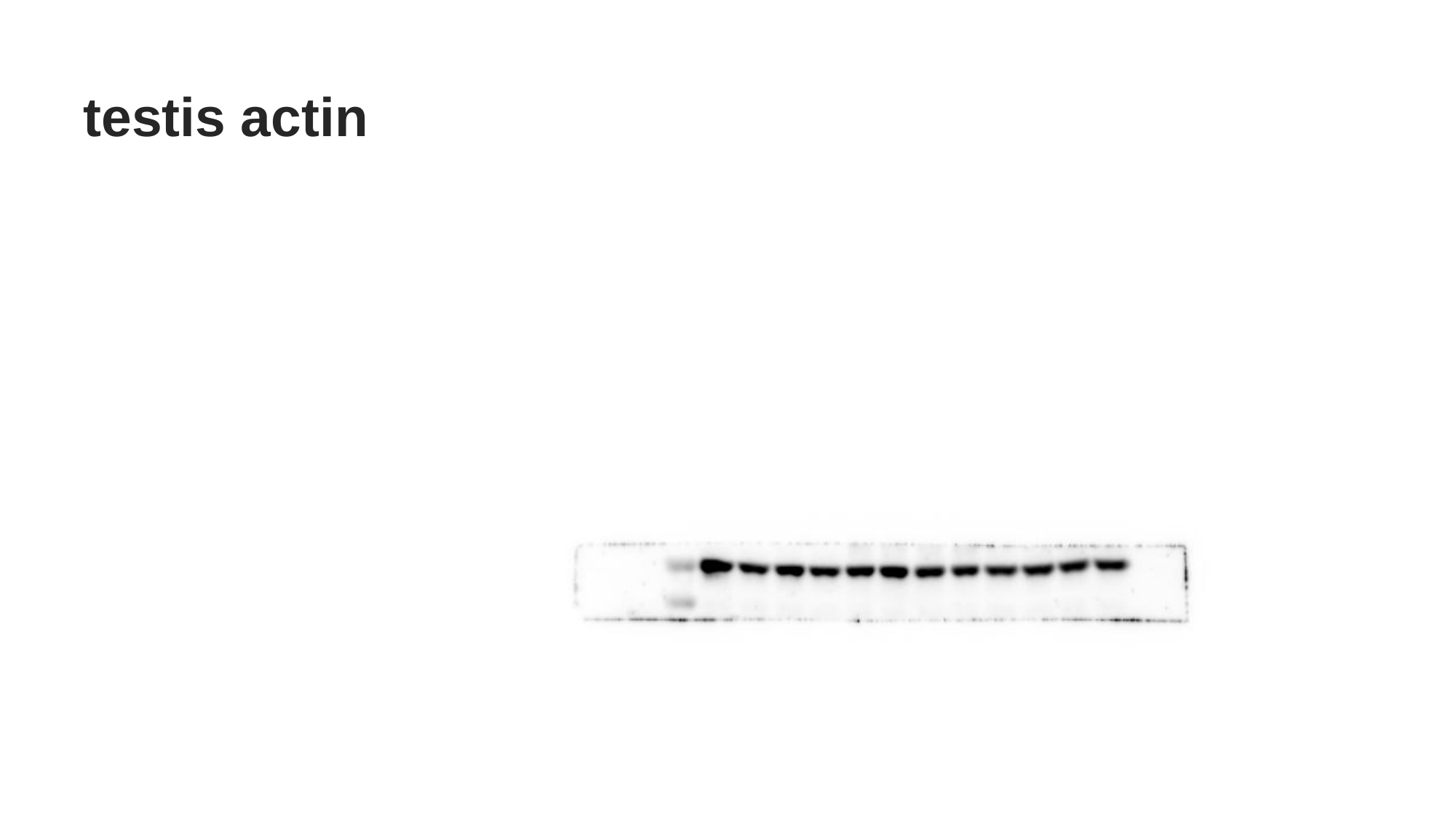

# testis actin

## Slide 18
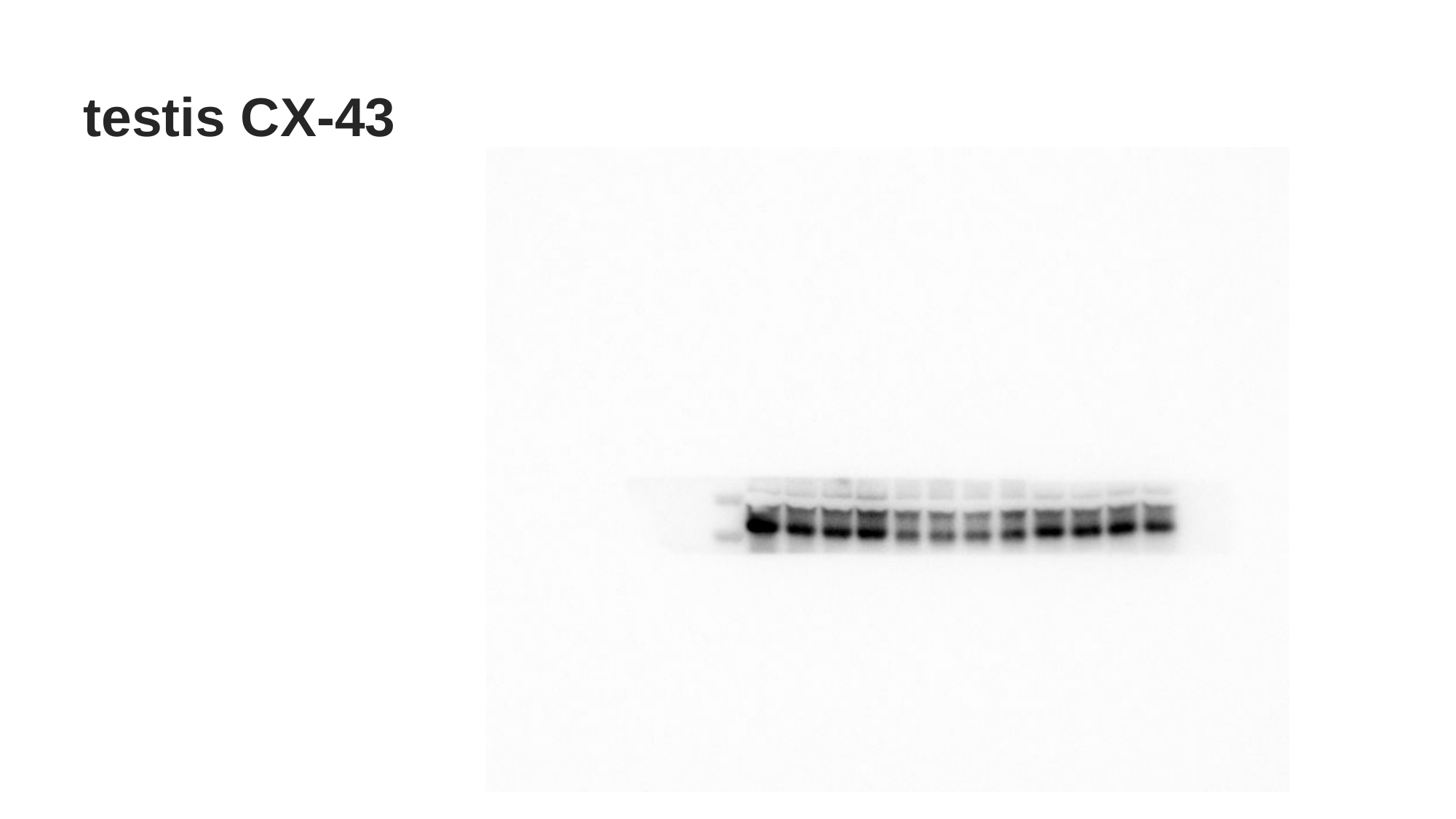

# testis CX-43

## Slide 19
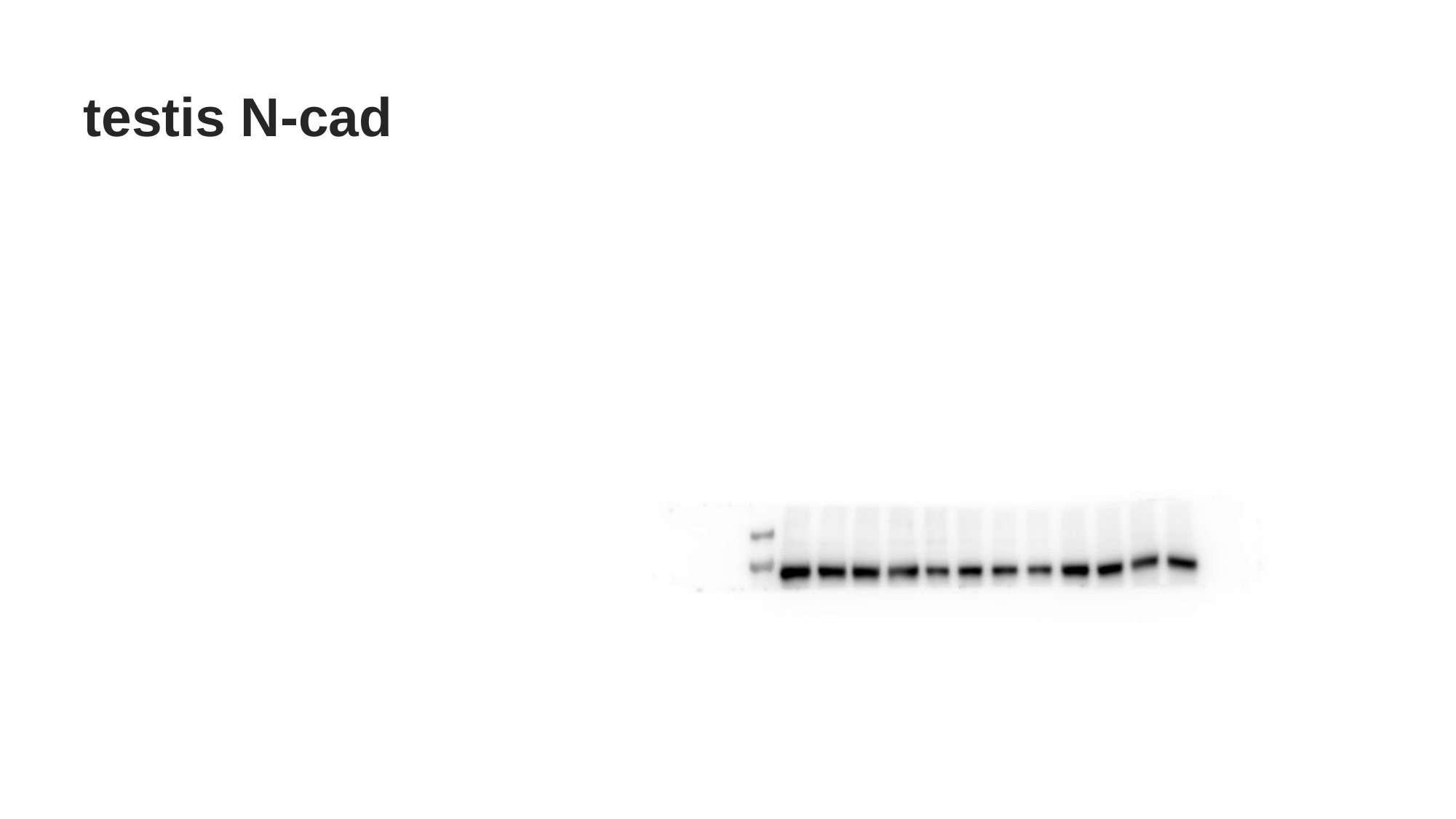

# testis N-cad

## Slide 20
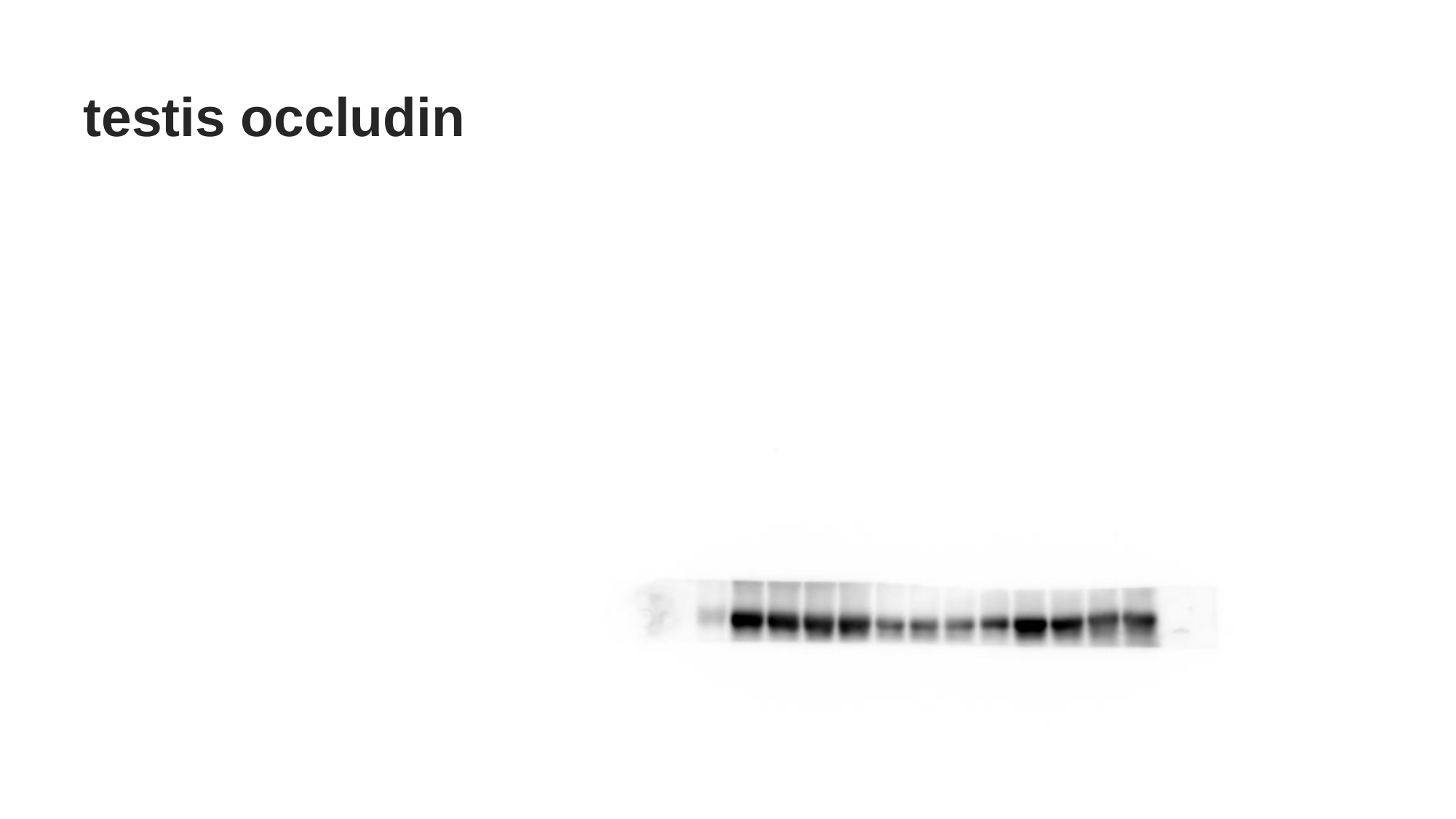

# testis occludin
